# Increased serum cholesterol and long-chain fatty acid levels are associated with the efficacy of nivolumab in patients with non-small cell lung cancer

**DOI:** 10.1007/s00262-021-02979-4

**Published:** 2021-06-05

**Authors:** Masato Karayama, Naoki Inui, Yusuke Inoue, Katsuhiro Yoshimura, Kazutaka Mori, Hironao Hozumi, Yuzo Suzuki, Kazuki Furuhashi, Tomoyuki Fujisawa, Noriyuki Enomoto, Yutaro Nakamura, Kazuhiro Asada, Tomohiro Uto, Masato Fujii, Takashi Matsui, Shun Matsuura, Dai Hashimoto, Mikio Toyoshima, Hideki Kusagaya, Hiroyuki Matsuda, Nao Inami, Yusuke Kaida, Mitsuru Niwa, Yasuhiro Ito, Takafumi Suda

**Affiliations:** 1 Department of Chemotherapy, Hamamatsu University School of Medicine, 1-20-1 Handayama, Hamamatsu, 431-3192 Japan; 2 Second Division, Department of Internal Medicine, Hamamatsu University School of Medicine, 1-20-1 Handayama, Hamamatsu, 431-3192 Japan; 3 Department of Clinical Pharmacology and Therapeutics, Hamamatsu University School of Medicine, 1-20-1 Handayama, Hamamatsu, 431-3192 Japan; 4 Department of Respiratory Medicine, Shizuoka City Shimizu Hospital, 1231 Miyakami, Shizuoka, 424-8636 Japan; 5 Department of Respiratory Medicine, Shizuoka General Hospital, 4-27-1 Kita-ando, Shizuoka, 420-0881 Japan; 6 Department of Respiratory Medicine, Iwata City Hospital, 513-2 Ohkubo, Iwata, 438-8550 Japan; 7 Department of Respiratory Medicine, Shizuoka City Hospital, 10-93 Ote-cho, Shizuoka, 420-8630 Japan; 8 Department of Respiratory Medicine, Seirei Mikatahara General Hospital, 3453 Mikatahara-cho, Hamamatsu, 433-8558 Japan; 9 Department of Respiratory Medicine, Fujieda Municipal General Hospital, 4-1-11 Surugadai, Fujieda, 426-8677 Japan; 10 Department of Respiratory Medicine, Seirei Hamamatsu General Hospital, 2-12-12 Sumiyoshi, Hamamatsu, 430-8558 Japan; 11 Department of Respiratory Medicine, Hamamatsu Rosai Hospital, 25 Shougen-cho, Hamamatsu, 430-8525 Japan; 12 Department of Respiratory Medicine, Shizuoka Saiseikai Hospital, 1-1-1 Oshika, Shizuoka, 422-8527 Japan; 13 Department of Respiratory Medicine, Japanese Red Cross Shizuoka Hospital, 8-2 Otemachi, Shizuoka, 420-0853 Japan; 14 Department of Respiratory Medicine, Ensyu Hospital, 1-1-1 Chuou, Hamamatsu, 430-0929 Japan; 15 Department of Respiratory Medicine, Hamamatsu Medical Center, 328 Tomitsuka-cho, Hamamatsu, 432-8580 Japan; 16 Department of Respiratory Medicine, National Hospital Organization Tenryu Hospital, 4201-2 Oro, Hamamatsu, 434-8511 Japan

**Keywords:** Anti-programmed death-1 therapy, Immunometabolism, Immune checkpoint inhibitor, Lipid, Metabolism

## Abstract

**Background:**

Lipids have immunomodulatory functions and the potential to affect cancer immunity.

**Methods:**

The associations of pretreatment serum cholesterol and long-chain fatty acids with the objective response rate (ORR), progression-free survival (PFS), and overall survival (OS) were evaluated in 148 patients with non-small cell lung cancer who received nivolumab.

**Results:**

When each lipid was separately evaluated, increased low-density lipoprotein (LDL)-cholesterol (*P* < 0.001), high-density lipoprotein (HDL)-cholesterol (*P* = 0.014), total cholesterol (*P* = 0.007), lauric acid (*P* = 0.015), myristic acid (*P* = 0.022), myristoleic acid (*P* = 0.035), stearic acid (*P* = 0.028), linoleic acid (*P* = 0.005), arachidic acid (*P* = 0.027), eicosadienoic acid (*P* = 0.017), dihomo-γ-linolenic acid (*P* = 0.036), and behenic acid levels (*P* = 0.032) were associated with longer PFS independent of programmed death ligand 1 (PD-L1) expression. Meanwhile, increased LDL-cholesterol (*P* < 0.001), HDL-cholesterol (*P* = 0.009), total cholesterol (*P* = 0.036), linoleic acid (*P* = 0.014), and lignoceric acid levels (*P* = 0.028) were associated with longer OS independent of PD-L1 expression. When multiple lipids were evaluated simultaneously, LDL-cholesterol (*P* = 0.003), HDL-cholesterol (*P* = 0.036), and lauric acid (*P* = 0.036) were independently predictive of PFS, and LDL-cholesterol (*P* = 0.008) and HDL-cholesterol (*P* = 0.031) were predictive of OS. ORR was not associated with any serum lipid.

**Conclusions:**

Based on the association of prolonged survival in patients with increased serum cholesterol and long-chain fatty acid levels, serum lipid levels may be useful for predicting the efficacy of immune checkpoint inhibitor therapy.

**Supplementary Information:**

The online version contains supplementary material available at 10.1007/s00262-021-02979-4.

## Introduction

Immune checkpoint inhibitors (ICIs) are increasingly used as new standard treatments for cancers, including non-small cell lung cancer (NSCLC) [1, 2]. However, only some patients benefit from ICI therapy, and the benefits are not sustained in most responders. To address unmet needs, novel biomarkers and/or combination therapies that can enhance the efficacy of ICIs have been intensively investigated [3, 4]. A better understanding of factors associated with the therapeutic benefits of ICIs could provide improved precision medicine and new insights into the mechanisms of response and resistance to ICIs.

Unlike cytotoxic chemotherapy with direct anti-tumor effects, ICIs induce anti-tumor responses via immune cells. Therefore, to understand the therapeutic effects of ICIs, assessments of host–tumor interactions are essential in addition to tumor characteristics. It has become increasingly evident that the functions of immune cells are strongly associated with metabolism. Specifically, metabolites have essential roles in controlling the function, differentiation, or longevity of immune cells, thereby affecting cancer immunity [5–11]. For examples, tryptophan and its metabolites are known to affect cancer immunity [12, 13]. Decreased tryptophan levels induce anergy in effector T cells, and increased levels of the tryptophan metabolite kynurenine result in regulatory T cell activation and attenuate anti-tumor immunity [12]. Previously, we found that decreased levels of 3-hydroxyanthranilic acid, a downstream metabolite of the tryptophan–kynurenine pathway, are associated with longer progression-free survival (PFS) in nivolumab-treated patients with NSCLC [14]. The evaluation of immunometabolism will provide important information for ICI therapy.

Lipids, which serve as energy resources and components of the cell membrane, have immunomodulatory potential. Lipids are necessary for immune cell activation, including effector T cells, natural killer T cells, macrophages, and dendritic cells [5–10]. Decreased cholesterol levels inhibited the proliferation and activation of T cells [15]. Inversely, increased cholesterol content fostered Th1 differentiation [16]. Lauric acid, a long-chain fatty acid, induced the polarization of naïve T cells toward Th1 and Th17 cells [17, 18], and it activated bone marrow-derived dendritic cells and increased T cell activation capacity [19]. Cancer cells require lipids for their proliferation and progression; therefore, these cells aggressively compete with immune cells for lipid uptake. Consequently, cancer cells deprive immune cells of these essential lipids and inhibit their activation [7, 20–24].

However, little is known about the roles of serum lipids in ICI efficacy. The influence of fatty acids on immunity differs by type. It is well known that ω-3 polyunsaturated fatty acids (PUFAs) promote anti-inflammatory responses, whereas ω-6 PUFAs and saturated fatty acids promote pro-inflammatory responses [19, 25–27]. Given the diverse immunoregulatory functions of lipids, we hypothesized that different lipids have different effects on the efficacy of ICI therapy. The current study assessed the pretreatment serum levels of multiple lipids and evaluated their associations with the efficacy of nivolumab in patients with previously treated NSCLC. In addition, we also evaluated pretreatment sera from patients with NSCLC who received cytotoxic chemotherapy.

## Patients and methods

### Study design

This was a post hoc analysis of a prospective, multicenter, observational study conducted in 14 hospitals in Japan between July 1, 2016, and December 11, 2018 [28]. Each patient provided written informed consent. Additionally, a retrospective cohort of patients who received cytotoxic chemotherapy (chemotherapy cohort) treated at Hamamatsu University Hospital between January 1, 2009, and December 31, 2019, was evaluated. The study followed the ethical standards of the Declaration of Helsinki. The study protocol was approved by the Institutional Review Board of Hamamatsu University School of Medicine (No. 19–083). The study was registered with the University Hospital Medical Information Network Clinical Trial Registry (000,039,188). This study followed the Strengthening the Reporting of Observational Studies in Epidemiology reporting guideline [29].

### Patients

The study inclusion criteria have been described in a previous publication [28]. Briefly, patients with an Eastern Cooperative Oncology Group performance status (ECOG-PS) of 0–2 who were scheduled to receive nivolumab monotherapy for previously treated advanced NSCLC were included. Patients lacking pretreatment serum samples were excluded. Response was assessed every four cycles by local investigators using Response Evaluation Criteria in Solid Tumors (RECIST) version 1.1.

The chemotherapy cohort included patients with advanced NSCLC who received first-line platinum-based chemotherapies. Patients who received non-platinum therapy or had histories of previous chemotherapy or ICI therapy were excluded. The clinical data were retrospectively evaluated via medical record review.

### Lipid measurements

Low-density lipoprotein (LDL)-cholesterol and high-density lipoprotein (HDL)-cholesterol levels were evaluated by the direct method (Choletest^®^ LDL and Choletest^®^ N HDL, respectively; SEKISUI Medical, Tokyo, Japan). Total cholesterol content was evaluated using an enzymatic method (Choletest^®^ CHO, SEKISUI Medical). Meanwhile, 24 long-chain fatty acids were evaluated via gas chromatography (GC-2010, Shimadzu, Kyoto, Japan): lauric acid, myristic acid, myristoleic acid, palmitic acid, palmitoleic acid, stearic acid, oleic acid, linoleic acid, γ-linolenic acid, linolenic acid, arachidic acid, eicosenoic acid, eicosadienoic acid, eicosatrienoic acid, dihomo-γ-linolenic acid, arachidonic acid, eicosapentaenoic acid, behenic acid, erucic acid, docosatetraenoic acid, docosapentaenoic acid, lignoceric acid, docosahexaenoic acid, and nervonic acid. Erucic acid was not included because its levels were below the limit of detection in most patients. All measurements were taken at a laboratory (SRL, Inc., Tokyo, Japan) certified by the College of American Pathologist and International Organization for Standardization 15,189.

### Evaluation of clinical features and outcomes

Clinical factors, including age, sex, smoking status, body mass index (BMI), pathology, clinical stage, ECOG-PS, and line of treatment, were recorded. Tumor-programmed death ligand-1 (PD-L1) protein expression was evaluated immunohistochemically using the tumor proportion score (TPS. E1L3N antibody (Cell Signaling Technology) was used for PD-L1 immunohistochemistry until Japanese approval of the 22C3 pharmDX assay (Agilent). The objective response rate (ORR, sum of the partial and complete response rates was evaluated using RECIST version 1.1.), PFS and overall survival (OS) were evaluated from the time of treatment initiation.

### Statistical analyses

Student’s *t* test and Fisher’s exact test were used for continuous and categorical variables, respectively. Pearson’s correlation analysis was used to assess the correlations between lipid levels and clinical factors. Kaplan–Meier analyses were used for PFS and OS. In these analyses, lipid levels were divided into three groups by concentration as follows: low (below the interquartile range [IQR]), intermediate (within the IQR), and high (exceeding the IQR). The log-rank trend test was used to examine the associations of PFS or OS with stepwise increases in lipid levels. Logistic regression analysis was used to evaluate predictive factors for ORR, and the Cox proportional hazard analysis was used to assess predictors of PFS and OS. Variables significant at *P* < 0.100 in univariate analyses were employed for multivariate analyses. Additionally, sex and ECOG-PS were associated with lipid levels; therefore, they were included in the multivariate analyses. Two types of multivariate analysis were performed using different combinations of variables. First, to evaluate predictive utility of a single lipid, each individual lipid was analyzed with adjustment for sex, ECOG-PS, and variables significant at *P* < 0.100 in univariate analysis (single-lipid analysis). Second, to identify major contributing factors among the lipids, multiple lipids significant at *P* < 0.100 in univariate analysis were analyzed together with adjustment for sex, ECOG-PS, and variables significant at *P* < 0.100 in univariate analysis (multiple-lipid analysis). When variables had strong correlations with each other (Pearson’s correlation coefficient > 0.7), only one was selected for the multiple-lipid analysis to avoid multicollinearity. Candidate combinations for multiple-lipid analysis were created by grouping lipids without strong correlations with each other. Among the candidate combinations, the best model based on the Akaike information criterion was selected as a representative combination of lipids (Supplementary Methods). *P* < 0.05 (two-sided) denoted significance. All values were analyzed using JMP v13.2.0 (SAS Institute Japan, Tokyo, Japan), excluding log-rank trend test data, which were analyzed using PRISM Version 7.0d (GraphPad Software, CA, USA).

## Results

### Patient characteristics

Among 200 patients who were enrolled in the original prospective study, 52 patients who did not have sufficient serum samples for lipid measurements were excluded. Therefore, 148 patients with assessable pretreatment serum samples were included in this post hoc analysis. Patient characteristics are presented in Table 1. Most patients were men (82.4%), and most had a history of smoking (88.5%) and ECOG-PS of 0–1 (94.6%). Ninety-three patients (62.8%) had non-squamous cell carcinoma. Tumor PD-L1 expression was assessed in 144 patients (97.3%), and the TPS was 1%–49% in 49 patients (33.1%) and ≥ 50% in 21 patients (14.2%). One hundred and thirty-eight patients (93.2%) received platinum-based therapies before nivolumab, and 82 (55.4%) received nivolumab as a second-line therapy. Overall ORR was 22.3%, and median PFS and OS were 3.3 (95% confidence interval [CI] = 2.1–5.4) and 14.8 months (95% CI = 12.8–19.7), respectively.

**Table 1:** Patient characteristics

	*N* = 148
Age, years	69 (63–74)
Sex, men	122 (82.4)
Smoking status, ever-smoker	131 (88.5)
Body mass index, kg/m^2^	21.2 (19.0–23.0)
Use of statins	20 (13.5)
ECOG-PS, 0/1/≥ 2	78 (52.7)/62 (41.9)/8 (5.4)
Stage, IIIb/IV/recurrence	33 (22.3)/101 (68.2)/14 (9.5)
Pathology, adeno/squamous/others	81 (54.7)/55 (37.1)/12 (8.1)
PD-L1 expression: TPS, < 1%/1–49%/≥ 50%/unknown	74 (50.0)/49 (33.1)/21 (14.2)/4 (2.7)
*EGFR* mutation, positive/wild-type/unknown	8 (5.4)/110 (74.3)/30 (20.3)
*ALK* fusion gene, positive/wild-type/unknown	1 (0.7)/110 (74.3)/37 (25.0)
Treatment line, 2nd/ ≥ 3rd	82 (55.4)/66 (44.6)

Data are expressed as the median (interquartile range) or number (%)

*ALK* anaplastic lymphoma kinase; *ECOG-PS* Eastern Cooperative Oncology Group performance status; *EGFR* epidermal growth factor receptor; *PD-L1* programmed death ligand-1; *TPS* tumor proportion score

### Associations of lipid levels with patient demographics

Women had significantly higher LDL-cholesterol (*P* = 0.005), HDL-cholesterol (*P* = 0.006), total cholesterol (*P* = 0.001), myristic acid (*P* = 0.042), palmitic acid (*P* = 0.008), stearic acid (*P* = 0.001), oleic acid (*P* = 0.014), linoleic acid (*P* < 0.001), γ-linolenic acid (*P* = 0.007), linolenic acid (*P* < 0.001), arachidic acid (*P* = 0.017), eicosadienoic acid (*P* = 0.016), eicosapentaenoic acid (*P* = 0.034), behenic acid (*P* = 0.014), docosapentaenoic acid (*P* < 0.001), and docosahexaenoic acid levels (*P* < 0.001, Supplementary Table 1). Patients with ECOG-PS of 0–1 had significantly higher LDL-cholesterol (*P* = 0.032), myristic acid (*P* = 0.021), myristoleic acid (*P* = 0.049), stearic acid (*P* = 0.038), linolenic acid (*P* = 0.042), dihomo-γ-linolenic acid (*P* = 0.013), behenic acid (*P* = 0.025), and lignoceric acid levels (*P* = 0.005) and significantly lower nervonic acid levels (*P* = 0.009) than those with ECOG-PS ≥ 2 (Supplementary Table 2). Lipid levels were not correlated with age or BMI (Supplementary Table 3). Strong correlations were noted between total cholesterol and LDL-cholesterol (*r* = 0.86), myristic acid and myristoleic acid (*r* = 0.88), palmitic acid and stearic acid (*r* = 0.92), palmitic acid and oleic acid (*r* = 0.94), oleic acid and stearic acid (*r* = 0.86), and lignoceric acid and behenic acid (*r* = 0.94). Correlations among other lipids are presented in Supplementary Table 3.

### Association of lipid levels with PFS

**Fig. 1:**
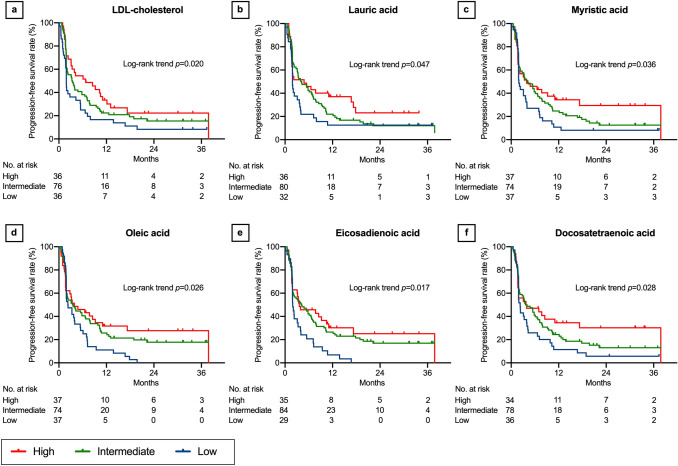
Progression-free survival after nivolumab therapy according to serum lipid levels: part 1. Low-density lipoprotein (LDL) cholesterol (**a**), lauric acid (**b**), myristic acid (**c**), oleic acid (**d**), eicosadienoic acid (**e**), and docosatetraenoic acid (**f**). Three concentration categories were created for each lipid: low (less than the interquartile range [IQR], blue line), intermediate (within the IQR, green line), and high (higher than the IQR, red line)

**Fig. 2:**
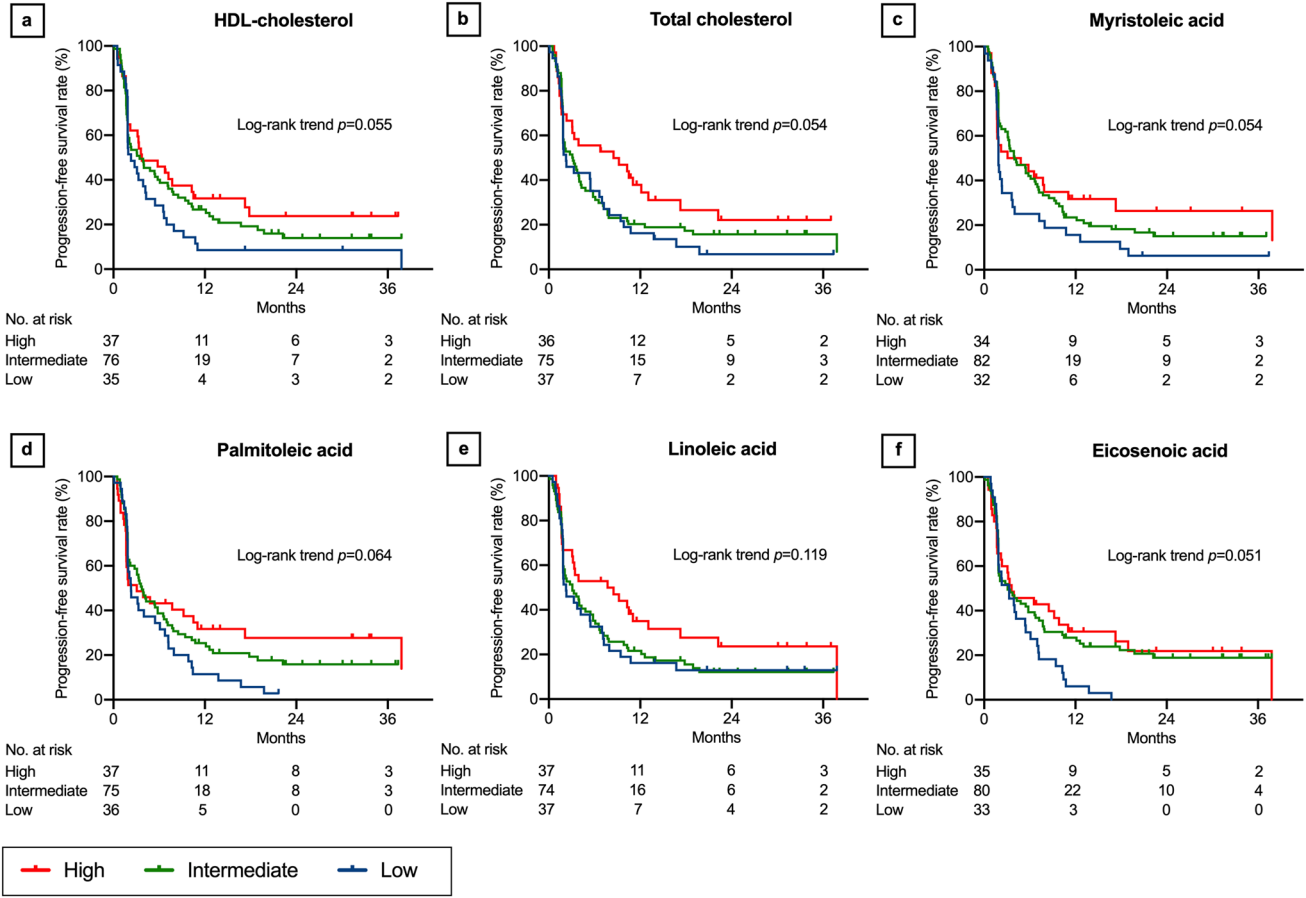
Progression-free survival after nivolumab therapy according to serum lipid levels: part 2. High-density lipoprotein (HDL) cholesterol (**a**), total cholesterol (**b**), myristoleic acid (**c**), palmitoleic acid (**d**), linoleic acid (**e**), and eicosenoic acid (**f**). Three concentration categories were created for each lipid: low (less than the interquartile range [IQR], blue line), intermediate (within the IQR, green line), and high (higher than the IQR, red line)

**Fig. 3:**
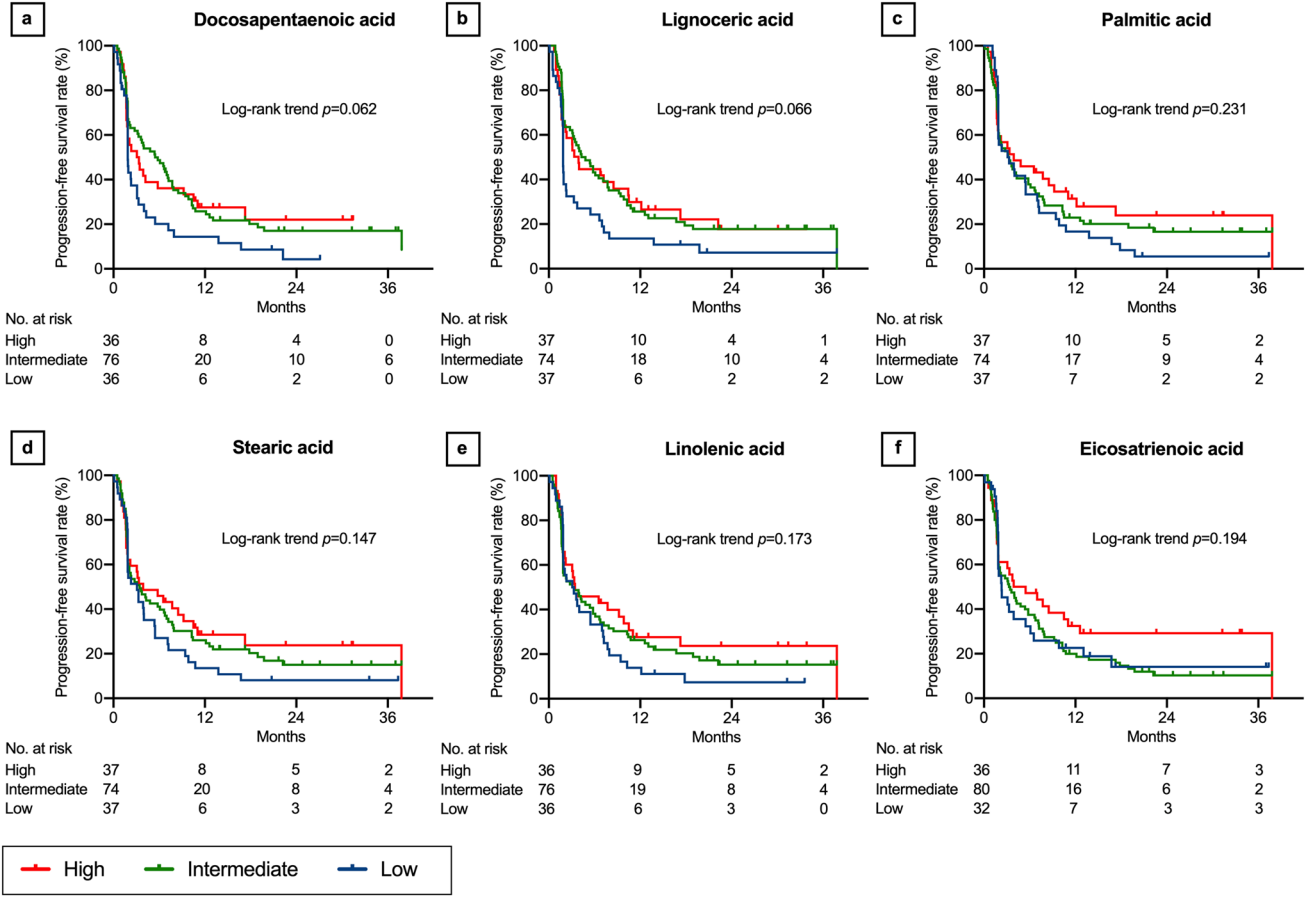
Progression-free survival after nivolumab therapy according to serum lipid levels: part 3. Docosapentaenoic acid (**a**), lignoceric acid (**b**), palmitic acid (**c**), stearic acid (**d**), linolenic acid (**e**), and eicosatrienoic acid (**f**). Three concentration categories were created for each lipid: low (less than the interquartile range [IQR], blue line), intermediate (within the IQR, green line), and high (higher than the IQR, red line)

**Fig. 4:**
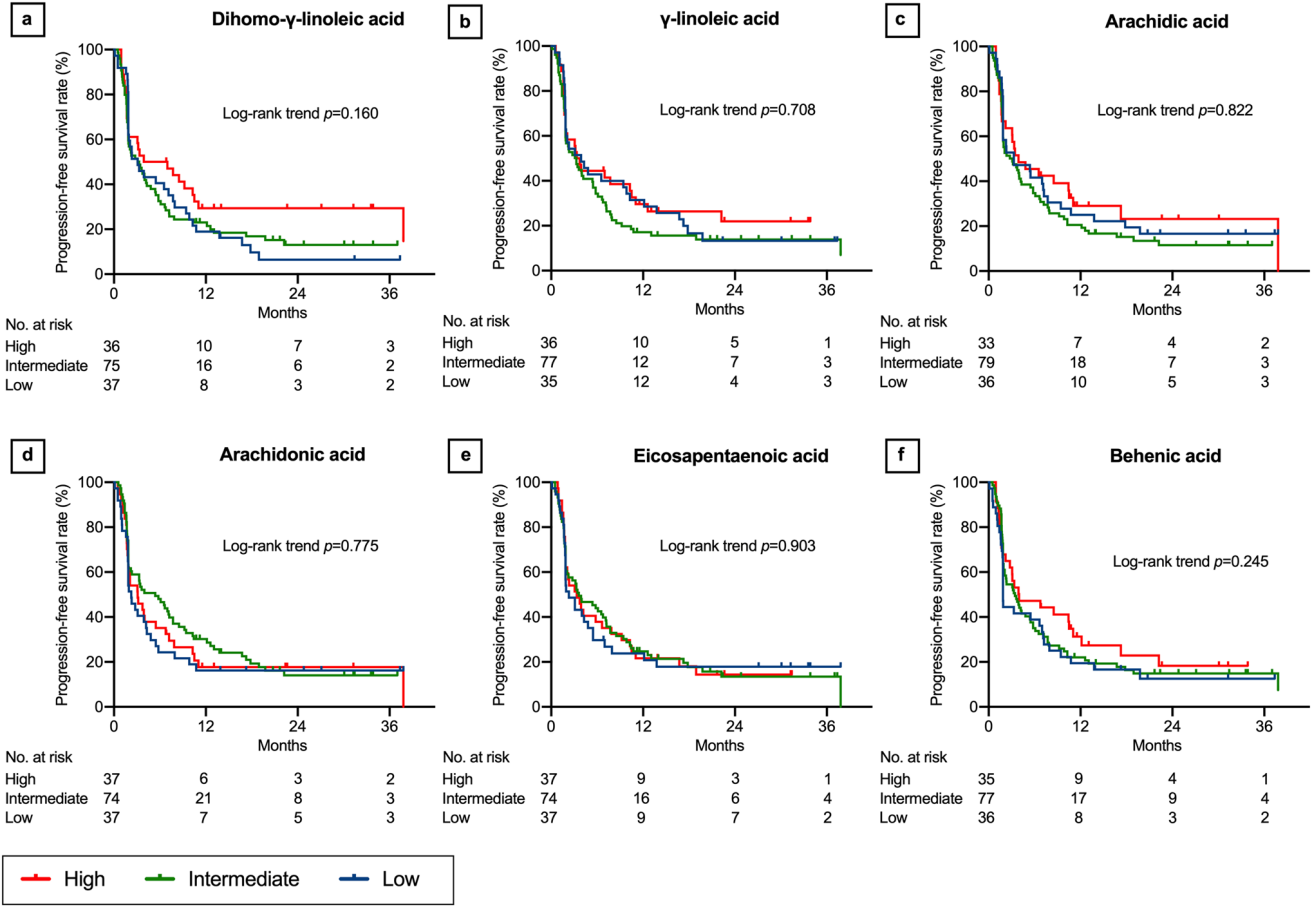
Progression-free survival after nivolumab therapy according to serum lipid levels: part 4. Dihomo-γ-linoleic acid (**a**), γ-linoleic acid (**b**), arachidic acid (**c**), arachidonic acid (**d**), eicosapentaenoic acid (**e**), and behenic acid (**f**). Three concentration categories were created for each lipid: low (less than the interquartile range [IQR], blue line), intermediate (within the IQR, green line), and high (higher than the IQR, red line). The Kaplan–Meier curves of docosahexaenoic acid and nervonic acid are presented in Supplementary Fig. 1

In the log-rank trend analyses of the Kaplan–Meier curves, PFS increased with increasing LDL-cholesterol (*P* = 0.020, Fig. 1a), lauric acid (*P* = 0.047, Fig. 1b), myristic acid (*P* = 0.036, Fig. 1c), oleic acid (*P* = 0.026, Fig. 1d), eicosadienoic acid (*P* = 0.017, Fig. 1e), and docosatetraenoic acid levels (*P* = 0.028, Fig. 1f). Although not statistically significant, PFS tended to increase with increasing HDL-cholesterol (*P* = 0.055, Fig. 2a), total cholesterol (*P* = 0.054, Fig. 2b), myristoleic acid (*P* = 0.054, Fig. 2c), palmitoleic acid (*P* = 0.064, Fig. 2d), linoleic acid (*P* = 0.119, Fig. 2e), eicosenoic acid (*P* = 0.051, Fig. 2f), docosapentaenoic acid (*P* = 0.062, Fig. 3a), lignoceric acid (*P* = 0.066, Fig. 3b), palmitic acid (*P* = 0.231, Fig. 3c), stearic acid (*P* = 0.147, Fig. 3d), linolenic acid (*P* = 0.173, Fig. 3e), eicosatrienoic acid (*P* = 0.194, Fig. 3f), and dihomo-γ-linoleic acid levels (*P* = 0.160, Fig. 4a). There was no association of PFS with γ-linoleic acid (*P* = 0.708, Fig. 4b), arachidic acid (*P* = 0.822, Fig. 4c), arachidonic acid (*P* = 0.775, Fig. 4d), eicosapentaenoic acid (*P* = 0.903, Fig. 4e), behenic acid (*P* = 0.245, Fig. 4f), docosahexaenoic acid (*P* = 0.481, Supplementary Fig. 1a), and nervonic acid level (*P* = 0.500, Supplementary Fig. 1b).

In univariate Cox proportional hazard analyses, increased LDL-cholesterol (*P* = 0.004), HDL-cholesterol (*P* = 0.019), total cholesterol (*P* = 0.042), lauric acid (*P* = 0.020), myristic acid (*P* = 0.026), myristoleic acid (*P* = 0.038), linoleic acid (*P* = 0.048), and eicosadienoic acid levels (*P* = 0.040) were predictive of longer PFS, similarly as smoking status, ECOG-PS, PD-L1 expression, and line of treatment (Supplementary Table 4). When each single lipid was analyzed with adjustment for sex, smoking status, ECOG-PS, PD-L1 expression, and line of treatment, LDL-cholesterol (*P* < 0.001), HDL-cholesterol (*P* = 0.014), total cholesterol (*P* = 0.007), lauric acid (*P* = 0.015), myristic acid (*P* = 0.022), myristoleic acid (*P* = 0.035), stearic acid (*P* = 0.028), linoleic acid (*P* = 0.005), arachidic acid (*P* = 0.027), eicosadienoic acid (*P* = 0.017), dihomo-γ-linolenic acid (*P* = 0.036), and behenic acid levels (*P* = 0.032) were predictive of PFS (Table 2). In multiple-lipid analysis, four combinations of lipids were selected according to their correlations (Supplementary Methods). When LDL-cholesterol, HDL-cholesterol, lauric acid, myristoleic acid, stearic acid, eicosenoic acid, and dihomo-γ-linolenic acid were analyzed together, LDL-cholesterol (*P* = 0.001), HDL-cholesterol (*P* = 0.027), and lauric acid levels (*P* = 0.040) were predictive of PFS (Table 2). In the other three combinations, no lipid was predictive (Supplementary Table 5).

**Table 2:** Multivariate Cox proportional hazard analyses of progression-free survival

	Single-lipid analysis		Multiple-lipid analysis	
Variables	Hazard ratio	*P*	Hazard ratio	*P*
LDL-cholesterol^a^	0.87 (0.82–0.93)	< 0.001	0.86 (0.78–0.94)	0.001
HDL-cholesterol^a^	0.81 (0.68–0.95)	0.014	0.83 (0.70–0.98)	0.027
Total cholesterol^a^	0.94 (0.90–0.98)	0.007		
Lauric acid^b^	0.80 (0.61–0.96)	0.015	0.80 (0.58–0.99)	0.040
Myristic acid^b^	0.89 (0.81–0.98)	0.022		
Myristoleic acid^c^	0.86 (0.76–0.99)	0.035	0.96 (0.80–1.13)	0.653
Palmitic acid^d^	0.93 (0.85–1.01)	0.100		
Palmitoleic acid^b^	0.96 (0.91–1.02)	0.209		
Stearic acid^d^	0.70 (0.51–0.96)	0.028	1.83 (0.95–3.46)	0.069
Oleic acid^d^	0.94 (0.87–1.02)	0.150		
Linoleic acid^d^	0.88 (0.81–0.96)	0.005		
γ-linolenic acid^c^	0.98 (0.95–1.01)	0.141		
Linolenic acid^c^	0.99 (0.97–1.00)	0.061		
Arachidic acid^c^	0.91 (0.84–0.99)	0.027		
Eicosenoic acid^c^	0.94 (0.87–1.02)	0.152	0.94 (0.84–1.06)	0.324
Eicosadienoic acid^c^	0.90 (0.83–0.98)	0.017		
Eicosatrienoic acid^c^	0.95 (0.87–1.04)	0.245		
Dihomo-γ-linolenic acid^c^	0.98 (0.97–0.99)	0.036	1.00 (0.98–1.02)	0.818
Arachidonic acid^b^	0.97 (0.94–1.01)	0.150		
Eicosapentaenoic acid^b^	0.99 (0.93–1.06)	0.867		
Behenic acid^c^	0.96 (0.92–0.99)	0.032		
Docosatetraenoic acid^c^	0.93 (0.84–1.02)	0.129		
Docosapentaenoic acid^c^	0.98 (0.95–1.01)	0.121		
Lignoceric acid^c^	0.96 (0.93–1.00)	0.072		
Docosahexaenoic acid^b^	0.96 (0.91–1.00)	0.080		
Nervonic acid^b^	0.89 (0.74–1.07)	0.227		

The hazard ratio was adjusted for sex, smoking status, Eastern Cooperative Oncology Group performance status, programmed death ligand-1 expression, and treatment line (2nd vs. ≥ 3rd). Total cholesterol, myristoleic acid, and linoleic acid were excluded from the multiple-lipid analysis because of their strong correlations with other lipids, and the results of the other combinations of the excluded lipids are presented in Supplementary Table 5

^a^per 10 mg/dL increase

^b^per 10 mg/dL increase

^c^per 1 mg/dL increase

^d^per 100 mg/dL increase

### Associations between lipids and OS

**Fig. 5:**
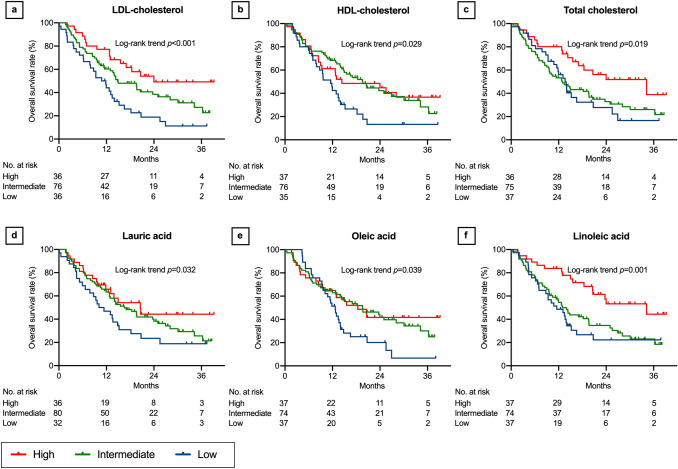
Overall survival after nivolumab therapy according to serum lipid levels: part 1. Low-density lipoprotein (LDL) cholesterol (**a**), high-density lipoprotein (HDL) cholesterol (**b**), total cholesterol (**c**), lauric acid (**d**), oleic acid (**e**), and linoleic acid (**f**). Three concentration categories were created for each lipid: low (less than the interquartile range [IQR], blue line), intermediate (within the IQR, green line), and high (higher than the IQR, red line)

**Fig. 6:**
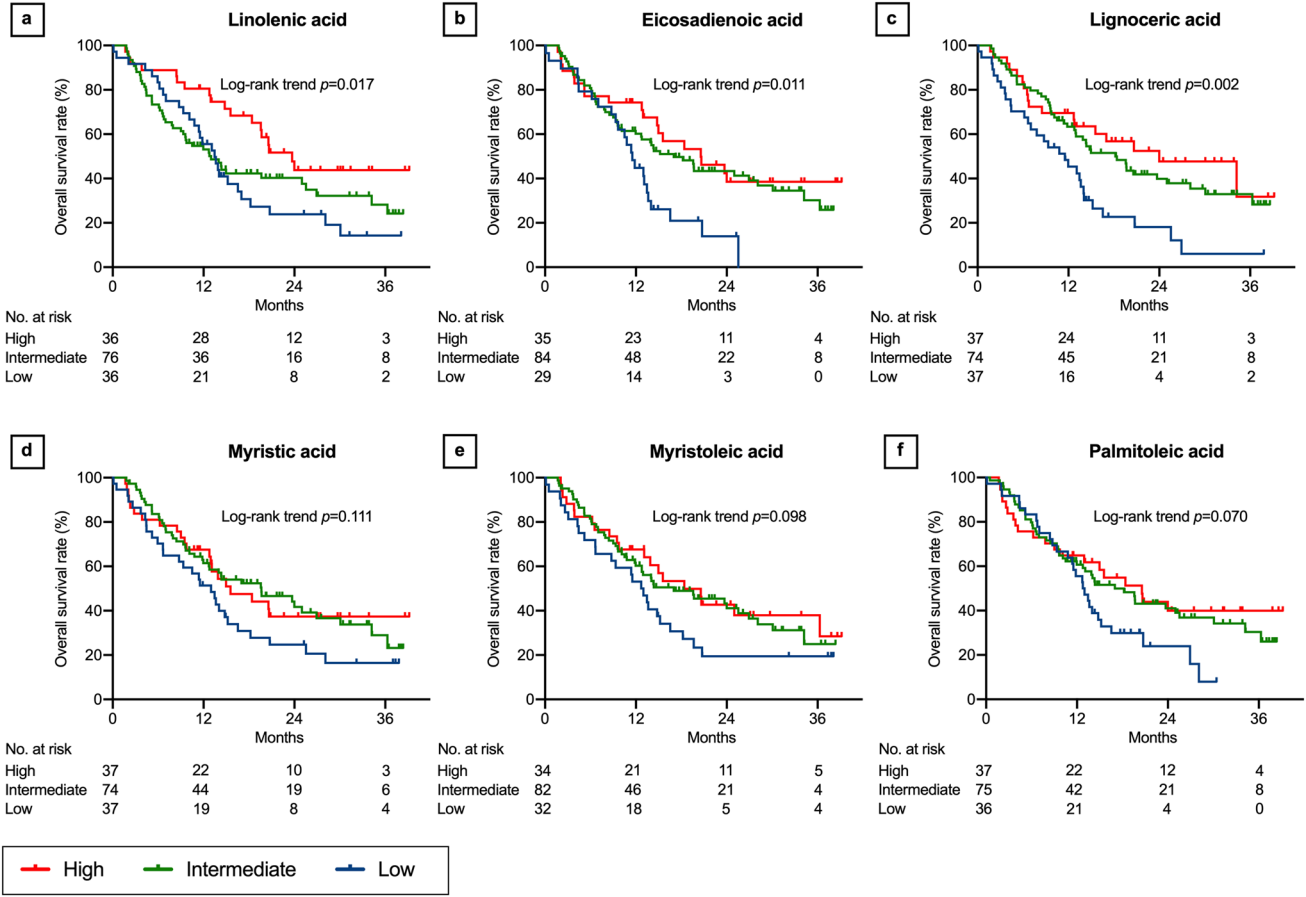
Overall survival after nivolumab therapy according to serum lipid levels: part 2. Linolenic acid (**a**), eicosadienoic acid (**b**), lignoceric acid (**c**), myristic acid (**d**), myristoleic acid (**e**), and palmitoleic acid (**f**). Three concentration categories were created for each lipid: low (less than the interquartile range [IQR], blue line), intermediate (within the IQR, green line), and high (higher than the IQR, red line)

**Fig. 7:**
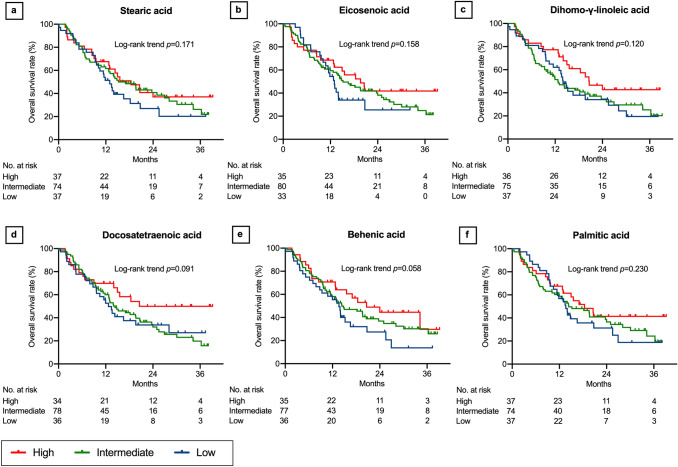
Overall survival after nivolumab therapy according to serum lipid levels: part 3. Stearic acid (**a**), eicosenoic acid (**b**), dihomo-γ-linoleic acid (**c**), docosatetraenoic acid (**d**), behenic acid (**e**), and palmitic acid (**f**). Three concentration categories were created for each lipid: low (less than the interquartile range [IQR], blue line), intermediate (within the IQR, green line), and high (higher than the IQR, red line)

**Fig. 8:**
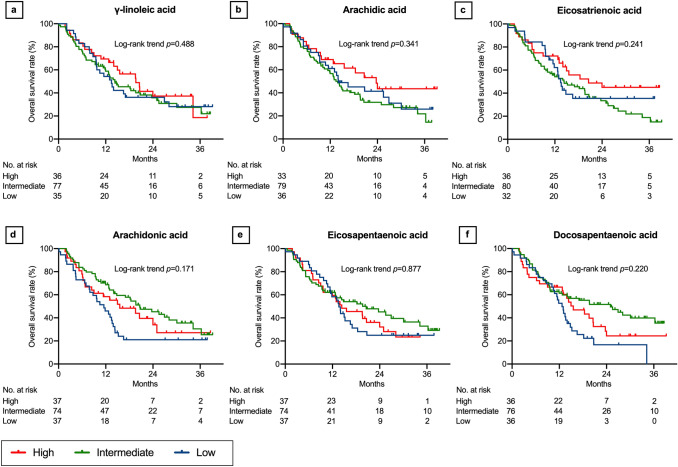
Overall survival after nivolumab therapy according to serum lipid levels: part 4. γ-linoleic acid (**a**), arachidic acid (**b**), eicosatrienoic acid (**c**), arachidonic acid (**d**), eicosapentaenoic acid (**e**), and docosapentaenoic acid (**f**). Three concentration categories were created for each lipid: low (less than the interquartile range [IQR], blue line), intermediate (within the IQR, green line), and high (higher than the IQR, red line). Kaplan–Meier curves of docosahexaenoic acid and nervonic acid are presented in Supplementary Fig. 2

In the log-rank trend analyses of the Kaplan–Meier curves, OS increased with increasing LDL-cholesterol (*P* < 0.001, Fig. 5a), HDL-cholesterol (*P* = 0.029, Fig. 5b), total cholesterol (*P* = 0.019, Fig. 5c), lauric acid (*P* = 0.032, Fig. 5d), oleic acid (*P* = 0.039, Fig. 5e), linoleic acid (*P* = 0.001, Fig. 5f), linolenic acid (*P* = 0.017, Fig. 6a), eicosadienoic acid (*P* = 0.011, Fig. 6b), and lignoceric acid levels (*P* = 0.002, Fig. 6c). Although not statistically significant, OS tended to increase with increasing myristic acid (*P* = 0.111, Fig. 6d), myristoleic acid (*P* = 0.098, Fig. 6e), palmitoleic acid (*P* = 0.070, Fig. 6f), stearic acid (*P* = 0.171, Fig. 7a), eicosenoic acid (*P* = 0.158, Fig. 7b), dihomo-γ-linolenic acid (*P* = 0.120, Fig. 7c), docosatetraenoic acid (*P* = 0.091, Fig. 7d), and behenic acid levels (*P* = 0.058, Fig. 7e). There was no association of OS with palmitic acid (*P* = 0.230, Fig. 7f), γ-linolenic acid (*P* = 0.488, Fig. 8a), arachidic acid (*P* = 0.341, Fig. 8b), eicosatrienoic acid (*P* = 0.241, Fig. 8c), arachidonic acid (*P* = 0.171, Fig. 8d), eicosapentaenoic acid (*P* = 0.877, Fig. 8e), docosapentaenoic acid (*P* = 0.220, Fig. 8f), docosahexaenoic acid (*P* = 0.348, Supplementary Fig. 2a), and nervonic acid levels (*P* = 0.130, Supplementary Fig. 2b).

In univariate Cox proportional hazard analyses, increased LDL-cholesterol (*P* < 0.001), HDL-cholesterol (*P* = 0.015), total cholesterol (*P* = 0.011), myristic acid (*P* = 0.025), myristoleic acid (*P* = 0.043), steric acid (*P* = 0.042), linoleic acid (*P* = 0.003), eicosadienoic acid (*P* = 0.016), behenic acid (*P* = 0.017), and lignoceric acid levels (*P* = 0.006) were predictive of OS, similarly as BMI, ECOG-PS, pathology, and PD-L1 expression (Supplementary Table 6). When each lipid was analyzed with adjustment for sex, BMI, ECOG-PS, pathology, and PD-L1 expression, LDL-cholesterol (*P* < 0.001), HDL-cholesterol (*P* = 0.009), total cholesterol (*P* = 0.036), linoleic acid (*P* = 0.014), and lignoceric acid levels (*P* = 0.028) were significant predictive factors (Table 3). In multiple-lipid analysis, nine combinations of lipids were selected according to their correlations (Supplementary Methods). When LDL-cholesterol, HDL-cholesterol, stearic acid, myristoleic acid, stearic acid, dihomo-γ-linolenic acid, and lignoceric acid were analyzed together, LDL-cholesterol (*P* = 0.005) and HDL-cholesterol (*P* = 0.035) were predictive of OS (Table 3). In the other eight combinations, LDL-cholesterol and HDL-cholesterol were predictive of OS (Supplementary Table 7).

**Table 3:** Multivariate cox proportional hazard analyses of overall survival

	Single-lipid analysis		Multiple-lipid analysis	
Variables	Hazard ratio	*P*	Hazard ratio	*P*
LDL-cholesterol^a^	0.87 (0.80–0.94)	< 0.001	0.86 (0.77–0.96)	0.005
HDL-cholesterol^a^	0.76 (0.62–0.93)	0.009	0.81 (0.66–0.99)	0.035
Total cholesterol^a^	0.95 (0.90–0.99)	0.036		
Lauric acid^b^	0.91 (0.68–1.07)	0.375		
Myristic acid^b^	0.93 (0.82–1.04)	0.251		
Myristoleic acid^c^	0.92 (0.79–1.05)	0.289	0.91 (0.75–1.11)	0.346
Palmitic acid^d^	0.98 (0.89–1.08)	0.678		
Palmitoleic acid^b^	1.00 (0.94–1.05)	0.916		
Stearic acid^d^	0.85 (0.58–1.23)	0.405	1.65 (0.90–3.01)	0.112
Oleic acid^d^	1.00 (0.92–1.09)	0.915		
Linoleic acid^d^	0.87 (0.79–0.97)	0.014		
γ-linolenic acid^c^	1.00 (0.96–1.03)	0.829		
Linolenic acid^c^	0.99 (0.98–1.01)	0.554		
Arachidic acid^c^	0.97 (0.88–1.06)	0.516		
Eicosenoic acid^c^	0.99 (0.89–1.08)	0.783		
Eicosadienoic acid^c^	0.92 (0.83–1.01)	0.105		
Eicosatrienoic acid^c^	0.98 (0.88–1.09)	0.759		
Dihomo-γ-linolenic acid^c^	0.99 (0.98–1.01)	0.441	1.00 (0.98–1.02)	0.831
Arachidonic acid^b^	0.97 (0.93–1.02)	0.296		
Eicosapentaenoic acid^b^	1.02 (0.95–1.08)	0.513		
Behenic acid^c^	0.96 (0.91–1.00)	0.086		
Docosatetraenoic acid^c^	0.96 (0.85–1.07)	0.479		
Docosapentaenoic acid^c^	1.00 (0.97–1.03)	0.810		
Lignoceric acid^c^	0.95 (0.90–0.99)	0.028	0.99 (0.93–1.06)	0.808
Docosahexaenoic acid^b^	0.98 (0.92–1.03)	0.400		
Nervonic acid^b^	1.06 (0.85–1.33)	0.581		

The hazard ratio was adjusted for sex, body mass index, Eastern Cooperative Oncology Group performance status, pathology, and programmed death ligand-1 expression. Total cholesterol, myristic acid, linoleic acid, and lignoceric acid were excluded from the multiple-lipid analysis because of their strong correlations with other lipids, and the results of the combination of the excluded lipids are presented in Supplementary Table 7

^a^per 10 mg/dL increase

^b^per 10 mg/dL increase

^c^per 1 mg/dL increase

^d^per 100 mg/dL increase

There was no significant association between lipid levels and ORR (Supplementary Table 8).

### Associations between lipid levels and the efficacy of cytotoxic chemotherapy

One hundred and thirteen patients in the chemotherapy cohort had comparable demographics as nivolumab-treated patients (Supplementary Table 9).

In univariate Cox proportional hazard analysis, total cholesterol (*P* = 0.044), linoleic acid (*P* = 0.018), eicosadienoic acid (*P* = 0.037), arachidonic acid (*P* = 0.046), and docosatetraenoic acid levels (*P* = 0.010) were predictive of PFS, similarly as age, ECOG-PS, and the receipt of cisplatin-based therapy (vs. carboplatin-based therapy, Supplementary Table 10). After adjustment for age, sex, ECOG-PS, and the receipt of cisplatin-based therapy, no lipids were significantly predictive of PFS (Supplementary Table 10). Univariate Cox proportional hazard analysis identified no predictive factors for OS, and univariate logistic regression analysis indicated that no factors were predictive of ORR (Supplementary Tables 11–12).

## Discussion

This was the first study to evaluate the association between multiple lipids and the efficacy of nivolumab in patients with previously treated NSCLC. Interestingly, we found that pretreatment serum cholesterol and long-chain fatty acid levels were significantly associated with PFS and OS, but not ORR, in patients treated with nivolumab, independent of ECOG-PS and PD-L1 expression. Conversely, no lipids were associated with the efficacy of cytotoxic chemotherapy. Serum lipid levels can be easily and noninvasively measured to assess patient status. In addition, lipids are natural products that can be administered easily and safely, and therefore, they have potential as therapeutic agents in combination with ICI therapy. Our data indicated the potential utility of lipids for predicting the efficacy of ICI therapy.

Increased serum lipid levels may compensate for lipid deprivation in immune cells in the tumor microenvironment. Cancer cells accumulate lipids in competition with immune cells, leading to in lipid deficiency in immune cells [7, 20–22]. It has been reported that increased exogenous lipids can potentially restore the activity of lipid-deficient immune cells in the tumor microenvironment. Mice with diet-induced hypercholesterolemia exhibited increased numbers of CD8+ and natural killer (NK) cells in hepatocellular carcinoma and adjacent tissues, leading to enhanced cytotoxic activity against cancer cells [30]. In patients with hepatocellular carcinoma, serum total cholesterol and LDL-cholesterol levels were positively correlated with the counts and cytotoxicity of intratumoral NK cells [30].

Another possible mechanism of the correlation between lipid levels and the efficacy of nivolumab was that cholesterol-induced T cell exhaustion can paradoxically increase the efficacy of anti-PD-1 therapy. It has been reported that increased extracellular cholesterol content induces immune checkpoint molecules on T cells including PD-1, leading to T cell exhaustion [31]. It is speculated that patients with cancer and elevated cholesterol levels exhibit PD-1-induced immune escape, increasing their susceptibility to anti-PD-1 therapy, whereas those without high cholesterol levels may experience immune escape caused by non-PD-1-induced pathways, leading to insusceptibility. Contrarily, cytotoxic chemotherapy does not confer any therapeutic advantage for PD-1-induced T cell exhaustion, and thus, this treatment did not influence the association between serum lipids and treatment efficacy.

Similar paradoxical effects were also reported in obesity, an established risk factor for several cancers. In 331 patients who received immunotherapy for melanoma, obese patients (BMI ≥ 30 kg/m^2^) exhibited significantly longer PFS and OS than those with normal BMI (18.5–24.9 kg/m^2^), but this was not replicated in patients who received chemotherapy [32]. Similar results were reported in 250 patients with a variety of cancers who received immunotherapies [33]. Mice with diet-induced obesity displayed increased PD-1-positive T cell exhaustion in the tumor microenvironment and greater tumor growth control diet-fed mice. Inversely, the efficacy of anti–PD-1 treatment was better in obese mice than in controls [33]. The study focused on increased leptin levels attributable to obesity as the cause of T cell exhaustion; however, the precise mechanisms underlying the improved efficacy of anti-PD-1 treatment in obesity were not clarified. The current study did not include any patients with BMI ≥ 30 kg/m^2^, and BMI was not associated with the levels of any lipids or the efficacy of nivolumab. However, obesity is often associated with hyperlipidemia, which can affect obesity-associated immune alterations.

Interestingly, ω-6 PUFAs (linoleic acid, arachidic acid, eicosadienoic acid, and dihomo-γ-linolenic acid) and saturated fatty acids (lauric acid, myristic acid, stearic acid, behenic acid, and lignoceric acid), which are known to promote pro-inflammatory response, displayed positive associations with treatment efficacy. On the contrary, ω-3 PUFAs (linolenic acid, eicosatetraenoic acid, eicosapentaenoic acid, docosapentaenoic acid, or docosahexaenoic acid), which are known to promote anti-inflammatory responses, exhibited no association with efficacy. Because of their counter-regulatory roles in immunity, ω-3 PUFAs are considered beneficial for inflammatory diseases, cardiovascular diseases, and cancers, whereas ω-6 PUFAs and saturated fatty acids are considered risk factors [17, 18, 34]. However, in the context of immune therapy, pro-inflammatory responses have potential benefits.

However, controversies exist regarding the roles of cholesterol and fatty acids in cancer immunity. Lipid cannot always provide favorable conditions for cancer immunity. It is reported that LDL-cholesterol inhibited T cell activation, resulting in impaired antitumor function [35]. Increased cholesterol and/or fatty acid levels have the potential to attenuate anticancer immunity via activation suppressor immune cells, such as regulatory T cells (Tregs), myeloid-derived suppressor cells (MDSCs), and immunosuppressive tumor-associated macrophages (TAMs) [20, 22, 24]. The difference in cancer immunity between LDL- and HDL-cholesterol is also unclear. Generally, LDL and HDL exert counter-regulatory effects on cholesterol transport: LDL promotes inflammation via cholesterol influx into immune cells, whereas HDL reduces inflammation via cholesterol efflux [8]. If cholesterol-induced pro-inflammatory responses are beneficial for anticancer immunity, then increased levels of HDL-cholesterol would be unfavorable. However, in the current study, increased HDL-cholesterol levels were associated with longer PFS and OS independent of LDL-cholesterol levels. The tumor microenvironment has complicated cross-interactions among tumor cells, effector cells, and regulatory immune cells, and metabolism is uniquely regulated in each cell type. Further studies are warranted to understand the complex mechanism of lipid metabolism in cancer immunity.

The current study had three main limitations. First, it is unknown which lipids have actual and/or the most relevant immunomodulatory activities that affect the efficacy of nivolumab. The multiple-lipid analysis using the selected combinations identified certain lipids as major contributing factors. However, selection based on correlation coefficients could not completely eliminate potential confounding. This is because lipids have a complicated metabolic cascade in which they are directly and/or indirectly associated with each other, and lipid composition varies among foods (major source of lipids for humans). In addition, it is also possible that selection based on correlations ignored the potential importance of the excluded lipids. Second, the differences in lipid composition and/or concentration between peripheral blood and the tumor microenvironment are unknown. In fact, cancer tissue is reported to have twofold higher free cholesterol and 3.5-fold higher esterified cholesterol levels, probably because of increased intake by tumor cells [36]. Third, serum lipids levels were provisionally determined according to the statistical distribution of the limited number of study patients. It is unknown whether higher serum levels will always lead to better clinical outcomes. In addition, it is also unknown whether increased lipid levels following supplemental treatment have beneficial effects. The regulation of lipid metabolism can potentially enhance the efficacy of immunotherapy, and this strategy has attracted attention as a novel therapeutic option [37–39]. Further studies are needed to evaluate the optimal levels and therapeutic benefits of lipids in ICI therapy.

In conclusion, increased serum cholesterol and long-chain fatty acid levels were associated with better PFS and OS in patients with previously treated NSCLC who received nivolumab. Serum lipids may be useful for predicting the efficacy of ICIs.

### Supplementary Information

Below is the link to the electronic supplementary material.

Supplementary file1 (PDF 1245 KB)

## Data Availability

The datasets used and/or analyzed during the current study are available from the corresponding author on reasonable request.
