# Supplementary material for: Increased serum cholesterol and long-chain fatty acid levels are associated with the efficacy of nivolumab in patients with non-small cell lung cancer
Source: Cancer Immunol Immunother. 2021 Jun 5;71(1):203–17. doi: 10.1007/s00262-021-02979-4 (PMC8738455; doi:10.1007/s00262-021-02979-4)
Supplement: Supplementary file 1 — Supplementary file1 (PDF 1245 KB) [file 262_2021_2979_MOESM1_ESM.pdf]

Supplementary Data

# Increased serum cholesterol and long-chain fatty acid levels are associated with the efficacy of nivolumab in patients with non-small-cell lung cancer

**Authors: Authors:** Masato Karayama, M.D., Ph.D., Naoki Inui, M.D., Ph.D., Yusuke Inoue, M.D., Ph.D., Katsuhiro Yoshimura, M.D., Ph.D., Kazutaka Mori, M.D., Ph.D., Hironao Hozumi, M.D., Ph.D., Yuzo Suzuki, M.D., Ph.D., Kazuki Furuhashi, M.D., Ph.D., Tomoyuki Fujisawa, M.D., Ph.D., Noriyuki Enomoto, M.D., Ph.D., Yutaro Nakamura, M.D., Ph.D., Kazuhiro Asada, M.D., Ph.D., Tomohiro Uto, M.D., Ph.D., Masato Fujii, M.D., Ph.D., Takashi Matsui, M.D., Ph.D., Shun Matsuura, M.D., Ph.D., Dai Hashimoto, M.D., Ph.D., Mikio Toyoshima, M.D., Ph.D., Hideki Kusagaya, M.D., Ph.D., Hiroyuki Matsuda, M.D., Ph.D., Nao Inami, M.D., Yusuke Kaida, M.D., Ph.D., Mitsuru Niwa, M.D., Ph.D., Yasuhiro Ito, M.D., Takafumi Suda, M.D., Ph.D.

Supplementary Data

## Supplementary Methods: Selection of lipids for multiple-lipid analysis.

LDL-cholesterol, HDL-cholesterol, total cholesterol, lauric acid, myristic acid, myristoleic acid, stearic acid, linoleic acid, eicosenoic acid, eicosadienoic acid, and dihomo-γ-linolenic acid were significantly associated with progression-free survival ($P < 0.100$) in univariate Cox proportional hazards analysis (Table 1). Meanwhile, LDL-cholesterol, HDL-cholesterol, total cholesterol, myristic acid, myristoleic acid, stearic acid, linoleic acid, eicosadienoic acid, and dihomo-γ-linolenic acid, behenic acid, and lignoceric acid were significantly associated with overall survival ($P < 0.100$) in univariate Cox proportional hazards analysis (Table 3). Among them, LDL-cholesterol was strongly correlated (Pearson correlation coefficient > 0.70) with total cholesterol and linoleic acid; total cholesterol was strongly correlated with linoleic acid, behenic acid, and lignoceric acid; myristic acid was strongly correlated with myristoleic acid; stearic acid was strongly correlated with linoleic acid, myristic acid, and eicosadienoic acid; linoleic acid was strongly correlated with stearic acid, eicosadienoic acid, behenic acid, and lignoceric acid' behenic acid was strongly correlated with lignoceric acid; and eicosadienoic acid was strongly correlated with dihomo-γ-linolenic acid (Supplementary Table 3). Lipids lacking strong correlations with each other were grouped as follows:

**PFS**:

**Combination A**: LDL-cholesterol, HDL-cholesterol, lauric acid, stearic acid, myristoleic acid, eicosenoic acid, and dihomo-γ-linolenic acid (AIC = 1022.5)

**Combination B**: total cholesterol, HDL-cholesterol, lauric acid, stearic acid, myristoleic acid, eicosenoic acid, and dihomo-γ-linolenic acid (AIC = 1031.7)

**Combination C**: HDL-cholesterol, lauric acid, myristic acid, eicosenoic acid, and dihomo-γ-linolenic acid (AIC = 1030.5);

**Combination D**: HDL-cholesterol, lauric acid, myristoleic acid, eicosenoic acid, and dihomo-γ-linolenic acid (AIC = 1030.1)

Combination A had the smallest AIC, and it was selected as the representative combination (Table 2). The results for Combinations B–D are presented in Supplementary Table 5.

**OS:**

**Combination A:** LDL-cholesterol, HDL-cholesterol, myristoleic acid, stearic acid, dihomo-γ-linolenic acid, and lignoceric acid (AIC = 769.2)

**Combination B**: LDL-cholesterol, HDL-cholesterol, myristoleic acid, stearic acid, dihomo-γ-linolenic acid, and behenic acid (AIC = 769.3)

Supplementary Data

**Combination C**: LDL-cholesterol, HDL-cholesterol, myristic acid, eicosadienoic acid, and lignoceric acid (AIC = 769.9)
**Combination D**: LDL-cholesterol, HDL-cholesterol, myristoleic acid, eicosadienoic acid, and lignoceric acid (AIC = 769.9)
**Combination E**: LDL-cholesterol, HDL-cholesterol, myristic acid, eicosadienoic acid, and behenic acid (AIC = 769.2)
**Combination F**: LDL-cholesterol, HDL-cholesterol, myristoleic acid, eicosadienoic acid, and behenic acid (AIC = 769.9)
**Combination G**: total cholesterol, HDL-cholesterol, stearic acid, myristoleic acid, and dihomo-γ-linolenic acid (AIC = 775.8)
**Combination H**: total cholesterol, HDL-cholesterol, myristic acid, and eicosadienoic acid (AIC = 774.0)
**Combination I**: total cholesterol, HDL-cholesterol, myristoleic acid, and eicosadienoic acid (AIC = 774.0)

Combination A had the smallest AIC, and it was selected as the representative combination (Table 3). The results for Combinations B–D are presented in Supplementary Table 7.

**Supplementary Figure 1. Progression-free survival after nivolumab therapy according to serum lipid levels (supplement to Figure 1-4).**

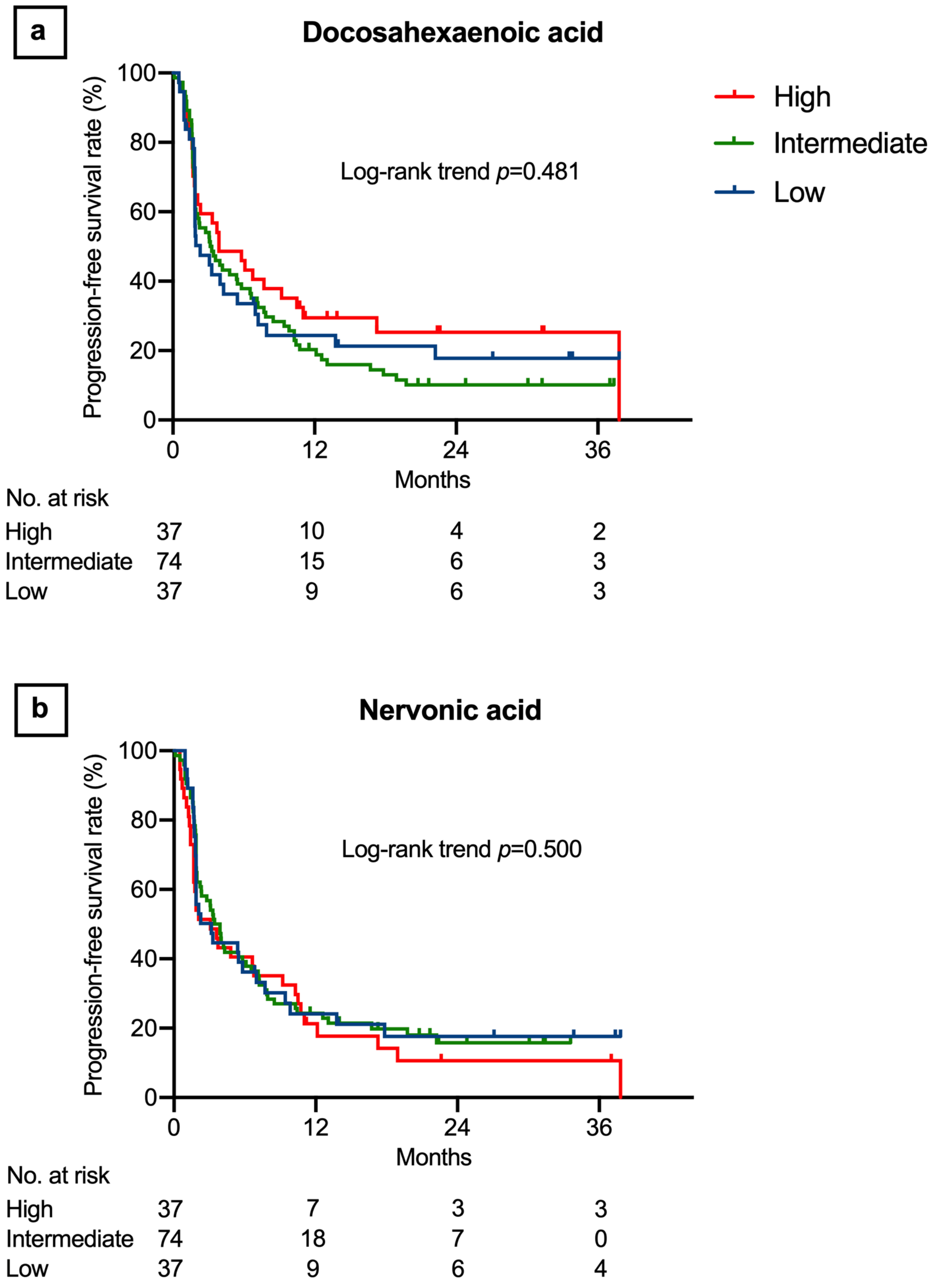

**Supplementary Figure 2. Overall survival after nivolumab therapy according to serum lipid levels (supplement to Figure 5-8).**

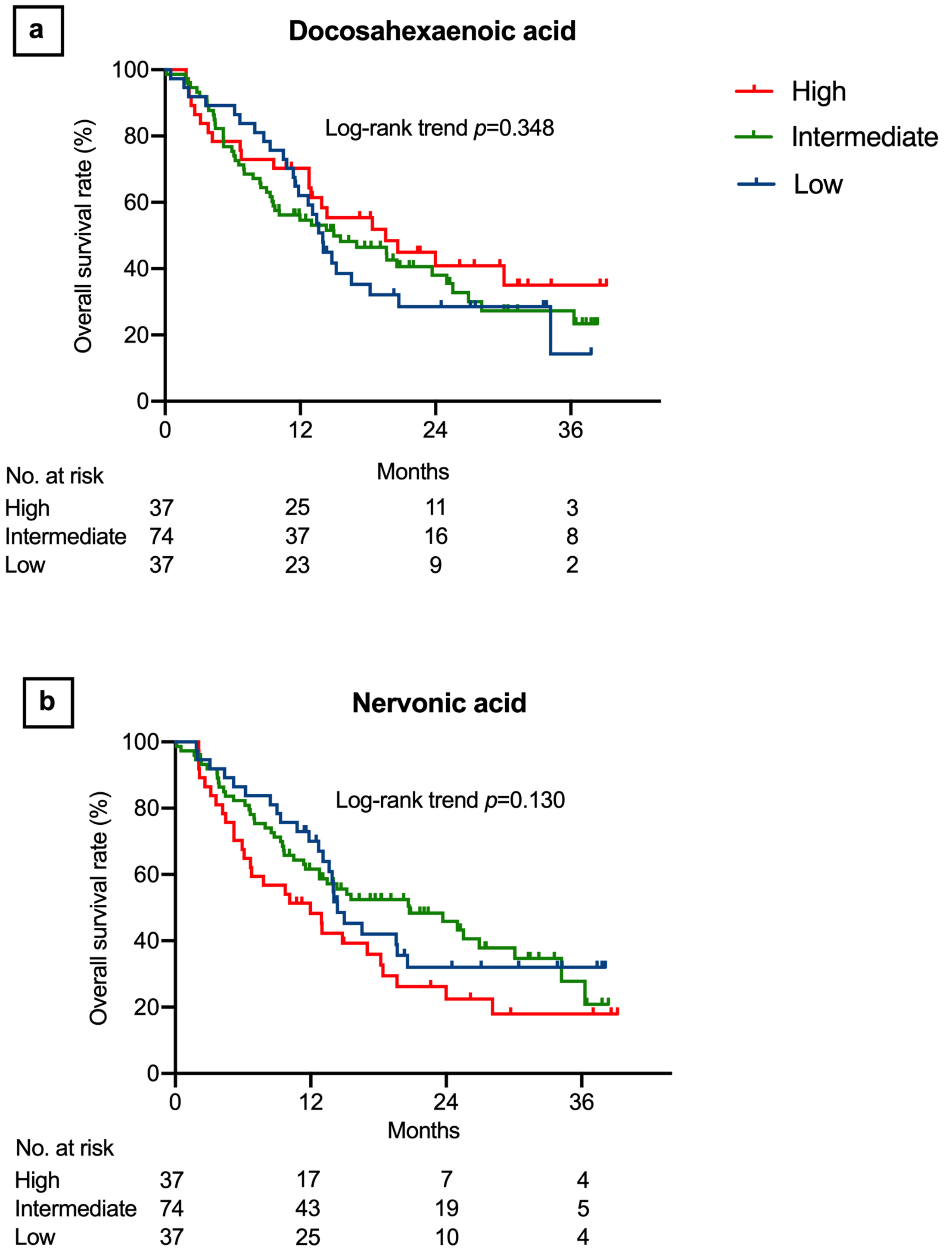

## Supplementary Table 1. Difference in lipid levels between the sexes

| Variables | All patients, n=148 | Men, n=122 | Women, n=26 | *p*-value |
|---|---|---|---|---|
| **LDL-cholesterol** | 85.0 (66.0 – 110.0) | 82.0 (64.0 − 108.0) | 106.0 (84.8 − 124.0) | 0.005 |
| **HDL-cholesterol** | 44.0 (38.0 – 51.8) | 43.0 (36.8 − 50.3) | 49.0 (43.0 − 56.5) | 0.006 |
| **Total cholesterol** | 182.0 (147.3 – 206.0) | 178.5 (144.8 − 204.0) | 199.0 (174.5 − 249.3) | 0.001 |
| **Lauric acid** | 2.2 (1.2 – 4.0) | 2.0 (1.2 − 4.0) | 2.9 (1.7 − 4.5) | 0.807 |
| **Myristic acid** | 21.1 (15.2 – 37.2) | 21.4 (13.7 − 33.6) | 32.8 (17.8 − 50.7) | 0.042 |
| **Myristoleic acid** | 1.4 (0.8 – 2.7) | 1.2 (0.8 − 2.5) | 1.9 (1.1 − 3.2) | 0.081 |
| **Palmitic acid** | 687.4 (568.9 –825.6) | 674.9 (548.5 − 816.2) | 751.7 (629.9 − 995.0) | 0.008 |
| **Palmitoleic acid** | 63.1 (45.9 – 82.8) | 61.9 (42.8 − 81.6) | 68.1 (49.6 − 90.9) | 0.098 |
| **Stearic acid** | 210.9 (173.4 – 252.0) | 202.6 (169.2 − 237.2) | 241.8 (209.9 − 306.2) | 0.001 |
| **Oleic acid** | 621.7 (502.4 – 790.4) | 602.3 (487.0 − 780.0) | 666.5 (577.1 – 970.3) | 0.014 |
| **Linoleic acid** | 755.2 (619.8 – 960.8) | 738.5 (602.7 − 934.3) | 907.9 (733.6 − 1108.3) | <0.001 |
| **γ-linolenic acid** | 7.1 (4.8 – 10.4) | 6.8 (4.7 − 10.0) | 8.5 (6.6 − 14.6) | 0.007 |
| **Linolenic acid** | 20.4 (15.0 – 32.6) | 19.1 (14.3 − 30.1) | 30.0 (20.2 − 44.9) | <0.001 |
| **Arachidic acid** | 7.9 (6.4 – 9.8) | 7.7 (6.2 − 9.6) | 8.6 (7.2 − 11.2) | 0.017 |
| **Eicosenoic acid** | 4.9 (3.9 – 6.6) | 4.9 (3.9 − 6.2) | 4.9 (4.0 − 7.0) | 0.094 |
| **Eicosadienoic acid** | 6.5 (5.3 – 8.2) | 6.4 (5.3 − 7.8) | 7.5 (5.8 − 9.4) | 0.016 |
| **Eicosatrienoic acid** | 2.1 (1.6 – 3.5) | 2.1 (1.6 − 3.4) | 2.5 (1.7 − 3.9) | 0.496 |
| **Dihomo-γ-linolenic acid** | 35.1 (27.3 – 43.2) | 33.9 (26.5 − 42.6) | 37.5 (33.6 − 53.0) | 0.079 |
| **Arachidonic acid** | 181.1 (144.7 – 207.1) | 175.8 (142.2 − 206.4) | 186.6 (172.0 − 220.0) | 0.063 |
| **Eicosapentaenoic aicd** | 44.0 (31.3 – 62.3) | 41.1 (29.6 − 59.8) | 62.8 (42.8 − 83.0) | 0.034 |
| **Behenic acid** | 17.3 (14.0 – 21.5) | 16.7 (13.4 − 21.0) | 20.9 (15.6 − 25.3) | 0.014 |
| **Docosatetraenoic acid** | 4.4 (3.5 – 5.7) | 4.3 (3.4 − 5.7) | 4.6 (3.8 − 6.8) | 0.446 |
| **Docosapentaenoic acid** | 14.3 (11.2 – 19.5) | 13.5 (10.7 − 18.2) | 19.8 (15.4 − 26.1) | <0.001 |
| **Lignoceric acid** | 16.3 (12.9 – 20.4) | 16.1 (12.5 − 19.4) | 17.9 (14.5 − 22.6) | 0.068 |

Supplementary Data

| | | | | |
|---|---|---|---|---|
| **Docosahexaenoic acid** | 117.2 (87.7 – 145.8) | 112.4 (82.0 − 138.5) | 147.4 (112.2 − 172.3) | <0.001 |
| **Nervonic acid** | 42.9 (36.0 – 49.4) | 42.8 (36.0 − 49.8) | 44.4 (36.0 − 49.4) | 0.888 |

Data are expressed as median (interquartile range). Data are expressed in μg/mL, except that total cholesterol, LDL-cholesterol, and HDL-cholesterol are in mg/dL.

HDL, high-density lipoprotein; LDL, low-density lipoprotein,

**Supplementary Table 2. Difference in lipids according to the Eastern Cooperative Oncology Group performance status**

| Variables | ECOG-PS 0-1, n=140 | ECOG-PS ≥2, n=8 | *p*-value |
|---|---|---|---|
| **LDL-cholesterol** | 88.0 (66.0 − 111.8) | 71.5 (51.8 − 74.0) | 0.032 |
| **HDL-cholesterol** | 39.5 (23.5 − 53.5) | 44.0 (38.0 − 51.8) | 0.138 |
| **Total cholesterol** | 183.5 (149.0 − 209.8) | 147.5 (132.0 − 189.5) | 0.078 |
| **Lauric acid** | 2.4 (1.4 − 4.2) | 1.0 (0.8 − 1.1) | 0.333 |
| **Myristic acid** | 22.8 (16.3 − 38.2) | 12.6 (10.7 − 15.9) | 0.021 |
| **Myristoleic acid** | 1.4 (0.9 − 2.7) | 0.7 (0.4 − 1.1) | 0.049 |
| **Palmitic acid** | 692.7 (570.0 − 843.6) | 628.6 (503.8 − 721.8) | 0.140 |
| **Palmitoleic acid** | 63.6 (46.0 − 82.8) | 47.5 (36.3 − 76.3) | 0.240 |
| **Stearic acid** | 216.2 (174.9 − 253.9) | 176.9 (147.9 − 199.0) | 0.038 |
| **Oleic acid** | 621.7 (502.4 − 795.8) | 597.5 (489.5 − 723.4) | 0.393 |
| **Linoleic acid** | 781.0 (623.2 − 969.8) | 626.5 (574.9 − 702.6) | 0.063 |
| **γ-linolenic acid** | 7.3 (4.9 − 10.9) | 4.8 (3.7 − 6.6) | 0.070 |
| **Linolenic acid** | 21.5 (15.1 − 33.8) | 15.5 (11.5 − 18.1) | 0.042 |
| **Arachidic acid** | 8.1 (6.4 − 9.8) | 6.6 (5.9 − 9.1) | 0.205 |
| **Eicosenoic acid** | 4.9 (3.9 − 6.6) | 5.4 (3.5 − 6.5) | 0.538 |
| **Eicosadienoic acid** | 6.7 (5.4 − 8.2) | 5.1 (4.7 − 6.2) | 0.068 |
| **Eicosatrienoic acid** | 2.3 (1.6 − 3.7) | 1.6 (1.1 − 1.9) | 0.077 |
| **Dihomo-γ-linolenic acid** | 35.5 (28.2 − 46.1) | 23.3 (18.1 − 29.3) | 0.013 |
| **Arachidonic acid** | 181.8 (149.2 − 208.6) | 144.1 (132.8 − 192.6) | 0.296 |
| **Eicosapentaenoic aicd** | 45.0 (31.1 − 62.3) | 35.5 (31.5 − 88.2) | 0.990 |
| **Behenic acid** | 17.3 (14.1 − 21.7) | 13.3 (12.6 − 17.0) | 0.025 |

| | | | |
|---|---|---|---|
| **Docosatetraenoic acid** | 4.4 (3.6 − 5.7) | 3.5 (2.8 − 4.7) | 0.102 |
| **Docosapentaenoic acid** | 14.3 (11.2 − 19.6) | 13.0 (11.5 − 14.9) | 0.276 |
| **Lignoceric acid** | 16.9 (13.3 − 20.5) | 12.4 (10.2 − 13.8) | 0.005 |
| **Docosahexaenoic acid** | 116.7 (84.2 − 146.3) | 124.6 (99.7 − 135.1) | 0.868 |
| **Nervonic acid** | 42.7 (35.9 − 47.9) | 52.9 (46.7 − 58.2) | 0.009 |

Data are expressed as median (interquartile range). Data are expressed in μg/mL, except that total cholesterol, LDL-cholesterol, and HDL-cholesterol are in mg/dL.

HDL, high-density lipoprotein; LDL, low-density lipoprotein,

## Supplementary Table 3: Correlations between lipid levels and clinical factors

| | BMI | TC | LDL-C | HDL-C | LauA | MA | MOA | PA | POA | SA | OA | LOA | GLA | LA |
|---|---|---|---|---|---|---|---|---|---|---|---|---|---|---|
| **Age** | -0.13 | -0.12 | -0.12 | -0.05 | -0.20 | -0.21 | -0.09 | -0.22 | -0.02 | -0.31 | -0.22 | -0.24 | -0.19 | -0.13 |
| **BMI** | | 0.10 | 0.16 | -0.08 | 0.03 | 0.28 | 0.24 | 0.28 | 0.13 | 0.25 | 0.28 | 0.30 | 0.18 | 0.33 |
| **TC** | | | 0.86 | 0.38 | 0.17 | 0.51 | 0.43 | 0.68 | 0.41 | 0.67 | 0.57 | 0.79 | 0.37 | 0.50 |
| **LDL-C** | | | | 0.25 | 0.19 | 0.48 | 0.40 | 0.54 | 0.30 | 0.56 | 0.43 | 0.70 | 0.36 | 0.49 |
| **HDL-C** | | | | | 0.05 | 0.11 | 0.06 | 0.14 | -0.04 | 0.21 | -0.02 | 0.28 | 0.25 | 0.09 |
| **LauA** | | | | | | 0.61 | 0.31 | 0.34 | 0.04 | 0.43 | 0.28 | 0.26 | 0.19 | 0.21 |
| **MA** | | | | | | | 0.88 | 0.84 | 0.52 | 0.81 | 0.74 | 0.63 | 0.54 | 0.57 |
| **MOA** | | | | | | | | 0.75 | 0.61 | 0.66 | 0.66 | 0.51 | 0.47 | 0.46 |
| **PA** | | | | | | | | | 0.76 | 0.92 | 0.94 | 0.79 | 0.62 | 0.64 |
| **POA** | | | | | | | | | | 0.60 | 0.80 | 0.44 | 0.52 | 0.44 |
| **SA** | | | | | | | | | | | 0.86 | 0.82 | 0.57 | 0.65 |
| **OA** | | | | | | | | | | | | 0.74 | 0.55 | 0.67 |
| **LOA** | | | | | | | | | | | | | 0.42 | 0.74 |
| **GLA** | | | | | | | | | | | | | | 0.40 |
| | **AraA** | **EEA** | **EDA** | **ETA** | **DGLA** | **AA** | **EPA** | **BA** | **DTA** | **DPA** | **LigA** | **DHA** | **NA** | |
| **Age** | -0.26 | -0.19 | -0.21 | -0.11 | -0.24 | -0.12 | 0.18 | -0.32 | -0.22 | -0.01 | -0.27 | 0.04 | 0.11 | |
| **BMI** | 0.26 | 0.23 | 0.26 | 0.07 | 0.31 | 0.08 | -0.04 | 0.21 | 0.14 | 0.11 | 0.13 | 0.12 | -0.22 | |
| **TC** | 0.73 | 0.44 | 0.64 | 0.31 | 0.53 | 0.46 | 0.28 | 0.78 | 0.36 | 0.51 | 0.78 | 0.49 | 0.42 | |
| **LDL-C** | 0.61 | 0.31 | 0.55 | 0.27 | 0.50 | 0.43 | 0.34 | 0.68 | 0.35 | 0.54 | 0.68 | 0.48 | 0.25 | |
| **HDL-C** | 0.16 | 0.00 | 0.13 | 0.14 | 0.19 | 0.25 | 0.16 | 0.30 | 0.10 | 0.16 | 0.34 | 0.21 | 0.04 | |
| **LauA** | 0.34 | 0.22 | 0.13 | 0.19 | 0.20 | 0.16 | 0.01 | 0.34 | 0.21 | 0.12 | 0.33 | 0.08 | -0.06 | |

| | | | | | | | | | | | | | | |
|---|---|---|---|---|---|---|---|---|---|---|---|---|---|---|
| **MA** | 0.61 | 0.59 | 0.64 | 0.52 | 0.62 | 0.34 | 0.12 | 0.51 | 0.62 | 0.51 | 0.48 | 0.32 | -0.07 | |
| **MOA** | 0.45 | 0.49 | 0.58 | 0.47 | 0.55 | 0.29 | 0.11 | 0.34 | 0.62 | 0.47 | 0.35 | 0.26 | -0.03 | |
| **PA** | 0.74 | 0.72 | 0.82 | 0.58 | 0.73 | 0.49 | 0.10 | 0.62 | 0.74 | 0.59 | 0.55 | 0.41 | 0.17 | |
| **POA** | 0.34 | 0.55 | 0.66 | 0.59 | 0.61 | 0.40 | 0.07 | 0.20 | 0.76 | 0.53 | 0.20 | 0.31 | 0.16 | |
| **SA** | 0.80 | 0.70 | 0.79 | 0.51 | 0.69 | 0.51 | 0.18 | 0.70 | 0.66 | 0.59 | 0.63 | 0.45 | 0.16 | |
| **OA** | 0.67 | 0.81 | 0.84 | 0.58 | 0.69 | 0.44 | -0.01 | 0.51 | 0.74 | 0.53 | 0.43 | 0.33 | 0.20 | |
| **LOA** | 0.82 | 0.63 | 0.83 | 0.31 | 0.62 | 0.42 | 0.07 | 0.80 | 0.48 | 0.45 | 0.76 | 0.41 | 0.27 | |
| **GLA** | 0.33 | 0.34 | 0.46 | 0.80 | 0.82 | 0.56 | 0.07 | 0.38 | 0.73 | 0.42 | 0.35 | 0.10 | -0.15 | |
| **LA** | 0.55 | 0.63 | 0.74 | 0.25 | 0.49 | 0.26 | 0.16 | 0.48 | 0.40 | 0.49 | 0.44 | 0.33 | 0.07 | |
| **AraA** | | 0.59 | 0.66 | 0.28 | 0.49 | 0.45 | 0.15 | 0.83 | 0.38 | 0.42 | 0.72 | 0.40 | 0.44 | |
| **EEA** | | | 0.77 | 0.39 | 0.48 | 0.30 | 0.02 | 0.39 | 0.52 | 0.37 | 0.31 | 0.30 | 0.23 | |
| **EDA** | | | | 0.44 | 0.72 | 0.34 | 0.02 | 0.55 | 0.64 | 0.48 | 0.50 | 0.36 | 0.23 | |
| **ETA** | | | | | 0.74 | 0.54 | -0.02 | 0.28 | 0.74 | 0.39 | 0.27 | 0.10 | -0.02 | |
| **DGLA** | | | | | | 0.53 | -0.01 | 0.52 | 0.77 | 0.44 | 0.49 | 0.17 | 0.00 | |
| **AA** | | | | | | | 0.14 | 0.42 | 0.67 | 0.38 | 0.42 | 0.41 | 0.33 | |
| **EPA** | | | | | | | | 0.11 | -0.08 | 0.72 | 0.17 | 0.62 | 0.15 | |
| **BA** | | | | | | | | | 0.34 | 0.34 | 0.94 | 0.27 | 0.28 | |
| **DTA** | | | | | | | | | | 0.43 | 0.31 | 0.18 | 0.01 | |
| **DPA** | | | | | | | | | | | 0.36 | 0.71 | 0.12 | |
| **LigA** | | | | | | | | | | | | 0.30 | 0.26 | |
| **DHA** | | | | | | | | | | | | | 0.34 | |

Data are expressed as Pearson correlation coefficient.

# Supplementary Data

LDL-C, low-density lipoprotein cholesterol; HDL-C, high-density lipoprotein cholesterol; TC, total cholesterol; LauA, lauric acid; MA, myristic acid; MOA, myristoleic acid; PA, palmitic acid; POA, palmitoleic acid; SA, stearic acid; OA, oleic acid, LOA, linoleic acid; GLA, γ-linolenic acid; LA, linolenic acid; AraA, arachidic acid; EEA, eicosenoic acid; EDA, eicosadienoic acid; ETA, eicosatrienoic acid; DGLA, dihomo-γ-linolenic acid; AA, arachidonic acid; EPA, eicosapentaenoic acid; BA, behenic acid; DTA, docosatetraenoic acid; DPA, docosapentaenoic acid; LigA, lignoceric acid; DHA, docosahexaenoic acid; NA, nervonic acid

**Supplementary Table 4. Univariate Cox proportional hazard analyses of progression-free survival**

| Variables | Hazard ratio | *p*-value |
|---|---|---|
| Age, per 10-year increase | 1.02 (0.84 − 1.23) | 0.866 |
| Sex, male | 0.62 (0.40 − 0.99) | 0.048 |
| Body-mass index, | 0.96 (0.90 − 1.02) | 0.169 |
| Smoking, ever-smoker | 0.52 (0.32 − 0.92) | 0.025 |
| Use of statins | 1.23 (0.72 − 1.97) | 0.434 |
| ECOG-PS, | | |
| 0 vs. 1 | 0.84 (0.58 − 1.21) | 0.341 |
| 0 vs. ≥2 | 0.42 (0.21 − 0.96) | 0.040 |
| 1 vs. ≥2 | 0.50 (0.25 − 1.15) | 0.098 |
| Pathology, squamous cell (vs. non-squamous) | 1.36 (0.94 − 1.95) | 0.105 |
| Stage, IIIb (vs. IV/ recurrent) | 0.85 (0.54 − 1.30) | 0.471 |
| PD-L1 expression (TPS), | | |
| 1 – 49% vs. <1% | 0.86 (0.58 – 1.26) | 0.444 |
| ≥50% vs. <1% | 0.54 (0.29 – 0.93) | 0.025 |
| ≥50% vs. 1 – 49% | 0.63 (0.33 – 1.12) | 0.117 |
| Treatment line, 2nd (vs. ≥3rd) | 1.55 (1.08 – 2.24) | 0.017 |
| LDL-cholesterol[a] | 0.91 (0.86 – 0.97) | 0.004 |
| HDL-cholesterol[a] | 0.83 (0.70 – 0.97) | 0.019 |
| Total cholesterol[a] | 0.96 (0.92 – 1.00) | 0.042 |
| Lauric acid[b] | 0.81 (0.62 – 0.97) | 0.020 |
| Myristic acid[b] | 0.90 (0.81 – 0.98) | 0.026 |
| Myristoleic acid[c] | 0.87 (0.76 – 0.98) | 0.038 |
| Palmitic acid[d] | 0.94 (0.86 – 1.02) | 0.165 |
| Palmitoleic acid[b] | 0.97 (0.92 – 1.03) | 0.348 |
| Stearic acid[d] | 0.77 (0.57 – 1.01) | 0.070 |

| | | |
|---|---|---|
| **Oleic acid[d]** | 0.96 (0.89 – 1.03) | 0.287 |
| **Linoleic acid[d]** | 0.92 (0.85 – 0.99) | 0.048 |
| **γ-linolenic acid[c]** | 0.98 (0.95 – 1.01) | 0.270 |
| **Linolenic acid[c]** | 0.99 (0.98 – 1.01) | 0.435 |
| **Arachidic acid[c]** | 0.94 (0.87 – 1.01) | 0.101 |
| **Eicosenoic acid[c]** | 0.93 (0.86 – 1.01) | 0.093 |
| **Eicosadienoic acid[c]** | 0.92 (0.85 – 0.99) | 0.040 |
| **Eicosatrienoic acid[c]** | 0.95 (0.86 – 1.03) | 0.243 |
| **Dihomo-γ-linolenic acid[c]** | 0.99 (0.98 – 1.00) | 0.071 |
| **Arachidonic acid[b]** | 0.99 (0.95 – 1.03) | 0.531 |
| **Eicosapentaenoic aid[b]** | 1.00 (0.95 – 1.05) | 0.945 |
| **Behenic acid[c]** | 0.97 (0.94 – 1.01) | 0.103 |
| **Docosatetraenoic acid[c]** | 0.94 (0.85 – 1.03) | 0.180 |
| **Docosapentaenoic acid[c]** | 0.99 (0.96 – 1.01) | 0.356 |
| **Lignoceric acid[c]** | 0.97 (0.93 – 1.01) | 0.116 |
| **Docosahexaenoic acid[b]** | 0.98 (0.94 – 1.02) | 0.363 |
| **Nervonic acid[b]** | 1.00 (0.84 – 1.20) | 0.981 |

ECOG-PS, Eastern Cooperative Oncology Group performance status; HDL, high-density lipoprotein; LDL, low-density lipoprotein; PD-L1, programmed cell death-ligand 1; TPS, tumor proportion score.

[a]per 10 mg/dL increase

[b]per 10 μg/mL increase

[c]per 1 μg/mL increase

[d]per 100 μg/mL increase

**Supplementary Table 5. Multivariate Cox proportional hazard analyses of progression-free survival: Other combinations in the multiple-lipid analyses**

| Variables | Combination B | | Combination C | | Combination D | |
|---|---|---|---|---|---|---|
| | Hazard ratio | *p*-value | Hazard ratio | *p*-value | Hazard ratio | *p*-value |
| **HDL-cholesterol[a]** | 0.84 (0.70 – 1.00) | 0.057 | 0.85 (0.71 – 1.01) | 0.061 | 0.84 (0.70 – 1.01) | 0.058 |
| **Total cholesterol[a]** | 0.96 (0.89 – 1.03) | 0.287 | | | | |
| **Lauric acid[b]** | 0.81 (0.59 – 1.01) | 0.058 | 0.84 (0.59 – 1.07) | 0.187 | 0.88 (0.67 – 1.04) | 0.156 |
| **Myristic acid[b]** | | | 1.02 (0.84 – 1.22) | 0.851 | | |
| **Myristoleic acid[c]** | 0.93 (0.77 – 1.10) | 0.411 | | | 0.95 (0.79 – 1.11) | 0.527 |
| **Stearic acid[d]** | 1.50 (0.76 – 2.92) | 0.242 | | | | |
| **Linoleic acid[d]** | | | 0.93 (0.82 – 1.07) | 0.316 | 0.94 (0.82 – 1.07) | 0.364 |
| **Eicosenoic acid[c]** | 0.97 (0.86 – 1.08) | 0.565 | 1.01 (0.90 – 1.12) | 0.866 | 1.02 (0.91 – 1.12) | 0.742 |
| **Dihomo-γ-linolenic acid[c]** | 0.99 (0.97 – 1.01) | 0.424 | 0.99 (0.98 – 1.01) | 0.442 | 1.00 (0.98 – 1.01) | 0.631 |

Hazard ratio was adjusted by sex, smoking status, ECOG-PS, PD-L1 expression, and treatment line (2nd vs. ≥3rd).

[a]per 10 mg/dL increase

[b]per 10 μg/mL increase

[c]per 1 μg/mL increase

[d]per 100 μg/mL increase

## Supplementary Table 6. Univariate Cox proportional hazard analyses of overall survival

| Variables | Hazard ratio | *p*-value |
|---|---|---|
| **Age, per 10-year increase** | 1.01 (0.82 – 1.27) | 0.898 |
| **Sex, male** | 0.85 (0.52 – 1.44) | 0.522 |
| **Body-mass index, per 1kg/m$^2$ increase** | 0.88 (0.82 – 0.95) | <0.001 |
| **Smoking, ever-smoker** | 0.73 (0.42 – 1.38) | 0.318 |
| **Use of statins** | 1.00 (0.52 – 1.77) | 0.991 |
| **ECOG-PS,** | | |
| **0 vs. 1** | 0.63 (0.42 – 0.97) | 0.035 |
| **0 vs. ≥2** | 0.28 (0.13 – 0.75) | 0.014 |
| **1 vs. ≥2** | 0.44 (0.20 – 1.17) | 0.095 |
| **Pathology, squamous cell (vs. non-squamous)** | 1.74 (1.14 – 2.63) | 0.010 |
| **Stage, IIIb (vs. IV/ recurrent)** | 0.80 (0.47 – 1.29) | 0.370 |
| **PD-L1 expression (TPS),** | | |
| **1 – 49% vs. <1%** | 1.00 (0.64 – 1.55) | 0.987 |
| **≥50% vs. <1%** | 0.49 (0.22 – 0.98) | 0.045 |
| **≥50% vs. 1 – 49%** | 0.49 (0.21 – 1.02) | 0.055 |
| **Treatment line, 2nd (vs. ≥3rd)** | 1.21 (0.81 – 1.83) | 0.350 |
| **LDL-cholesterol[a]** | 0.88 (0.82 − 0.95) | <0.001 |
| **HDL-cholesterol[a]** | 0.79 (0.65 − 0.95) | 0.015 |
| **Total cholesterol[a]** | 0.94 (0.90 − 0.98) | 0.011 |
| **Lauric acid[b]** | 0.85 (0.61 – 1.03) | 0.200 |
| **Myristic acid[b]** | 0.88 (0.78 – 0.98) | 0.025 |
| **Myristoleic acid[c]** | 0.86 (0.73 – 0.98) | 0.043 |
| **Palmitic acid[d]** | 0.93 (0.85 – 1.01) | 0.115 |
| **Palmitoleic acid[b]** | 0.97 (0.92 – 1.03) | 0.354 |
| **Stearic acid[d]** | 0.71 (0.51 – 0.98) | 0.042 |

| | | |
|---|---|---|
| **Oleic acid[d]** | 0.96 (0.88 – 1.03) | 0.259 |
| **Linoleic acid[d]** | 0.87 (0.80 – 0.95) | 0.003 |
| **γ-linolenic acid[c]** | 0.98 (0.95 – 1.01) | 0.250 |
| **Linolenic acid[c]** | 0.99 (0.98 – 1.00) | 0.232 |
| **Arachidic acid[c]** | 0.93 (0.86 – 1.01) | 0.100 |
| **Eicosenoic acid[c]** | 0.93 (0.84 – 1.01) | 0.112 |
| **Eicosadienoic acid[c]** | 0.89 (0.81 – 0.97) | 0.016 |
| **Eicosatrienoic acid[c]** | 0.94 (0.84 – 1.03) | 0.220 |
| **Dihomo-γ-linolenic acid[c]** | 0.99 (0.97 – 1.00) | 0.056 |
| **Arachidonic acid[b]** | 0.97 (0.92 – 1.01) | 0.181 |
| **Eicosapentaenoic aid[b]** | 1.02 (0.96 – 1.09) | 0.465 |
| **Behenic acid[c]** | 0.95 (0.91 – 0.99) | 0.017 |
| **Docosatetraenoic acid[c]** | 0.92 (0.83 – 1.02) | 0.128 |
| **Docosapentaenoic acid[c]** | 1.00 (0.97 – 1.03) | 0.999 |
| **Lignoceric acid[c]** | 0.94 (0.90 – 0.98) | 0.006 |
| **Docosahexaenoic acid[b]** | 0.98 (0.93 – 1.03) | 0.384 |
| **Nervonic acid[b]** | 1.17 (0.96 – 1.44) | 0.120 |

ECOG-PS, Eastern Cooperative Oncology Group performance status; HDL, high-density lipoprotein; LDL, low-density lipoprotein; PD-L1, programmed cell death-ligand 1; TPS, tumor proportion score.

[a]per 10 mg/dL increase

[b]per 10 μg/mL increase

[c]per 1 μg/mL increase

[d]per 100 μg/mL increase

**Supplementary Table 7. Multivariate Cox proportional hazard analyses of overall survival: Other combinations in the multiple-lipid analyses**

| Variables | Combination B | | Combination C | | Combination D | | Combination E | |
|---|---|---|---|---|---|---|---|---|
| | Hazard ratio | *p*-value | Hazard ratio | *p*-value | Hazard ratio | *p*-value | Hazard ratio | *p*-value |
| **LDL-cholesterol[a]** | 0.85 (0.76 – 0.95) | 0.003 | 0.88 (0.78 – 0.98) | 0.017 | 0.88 (0.78 – 0.98) | 0.018 | 0.86 (0.77 – 0.96) | 0.008 |
| **HDL-cholesterol[a]** | 0.81 (0.65 – 0.98) | 0.030 | 0.81 (0.65 – 0.99) | 0.040 | 0.81 (0.65 – 0.99) | 0.039 | 0.80 (0.65 – 0.98) | 0.034 |
| **Myristic acid[b]** | | | 1.02 (0.88 – 1.18) | 0.784 | | | 1.01 (0.87 – 1.16) | 0.861 |
| **Myristoleic acid[c]** | 0.92 (0.74 – 1.10) | 0.374 | | | 1.01 (0.85 – 1.17) | 0.935 | | |
| **Stearic acid[d]** | 1.61 (0.83 – 3.04) | 0.158 | | | | | | |
| **Eicosadienoic acid[c]** | | | 0.99 (0.88 – 1.12) | 0.957 | 1.00 (0.88 – 1.12) | 0.981 | 0.99 (0.87 – 1.11) | 0.870 |
| **Dihomo-γ-linolenic acid[c]** | 1.00 (0.98 – 1.02) | 0.837 | | | | | | |
| **Behenic acid[c]** | 1.00 (0.98 – 1.02) | 0.997 | | | | | 1.03 (0.96 – 1.09) | 0.382 |
| **Lignoceric acid[c]** | | | 1.01 (0.95 – 1.08) | 0.722 | 1.01 (0.95 – 1.07) | 0.681 | | |
| | **Combination F** | | **Combination G** | | **Combination H** | | **Combination I** | |
| **LDL-cholesterol[a]** | 0.87 (0.78 – 0.96) | 0.008 | | | | | | |
| **HDL-cholesterol[a]** | 0.80 (0.65 – 0.98) | 0.033 | 0.80 (0.64 – 0.99) | 0.039 | 0.78 (0.62 – 0.97) | 0.026 | 0.78 (0.62 – 0.97) | 0.026 |
| **Total cholesterol[a]** | | | 0.95 (0.88 – 1.03) | 0.188 | 0.99 (0.92 – 1.06) | 0.704 | 0.99 (0.92 – 1.06) | 0.693 |
| **Myristic acid[b]** | | | | | 0.99 (0.85 – 1.13) | 0.877 | | |

| | | | | | | | | |
|---|---|---|---|---|---|---|---|---|
| **Myristoleic acid[c]** | 1.01 (0.85 – 1.17) | 0.915 | 0.92 (0.74 – 1.09) | 0.365 | | | 0.98 (0.82 – 1.15) | 0.853 |
| **Stearic acid[d]** | | | 1.42 (0.75 – 2.58) | 0.274 | | | | |
| **Eicosadienoic acid[c]** | 0.99 (0.87 – 1.11) | 0.889 | | | 0.95 (0.83 – 1.07) | 0.439 | 0.95 (0.84 – 1.07) | 0.437 |
| **Dihomo-γ-linolenic acid[c]** | | | 1.00 (0.98 – 1.02) | 0.861 | | | | |
| **Behenic acid[c]** | 1.03 (0.97 – 1.09) | 0.355 | | | | | | |

Hazard ratio was adjusted by clinical factors including sex, body-mass index, ECOG-PS, pathology, and PD-L1 expression. Total cholesterol, myristoleic acid, linoleic acid, and lignoceric acid were excluded for multiple lipids analysis in Table 3, because there were strong correlations between LDL-cholesterol and total cholesterol (r=0.86), myristic acid and myristoleic acid (r=0.88), myristic acid and stearic acid (r=0.81), linoleic acid and steric acid (r=0.82), linoleic acid and eicosadienoic acid (r=0.83), linoleic acid and behenic acid (r=0.80), and lignoceric acid and behenic acid (r=0.94). Combination A employed total cholesterol (instead of LDL-cholesterol), Combination B employed myristic acid, linoleic acid, and lignoceric acid (instead of myristoleic acid, stearic acid, eicosadienoic acid and behenic acid), and Combination C employed total cholesterol, myristic acid, linoleic acid, and lignoceric acid (instead of LDL-cholesterol, myristoleic acid, stearic acid, eicosadienoic acid and behenic acid).

[a]per 10 mg/dL increase

[b]per 10 μg/mL increase

[c]per 1 μg/mL increase

[d]per 100 μg/mL increase

## Supplementary Table 8. Univariate logistic regression analyses of objective response

| Variables | Odds ratio | *p*-value |
|---|---|---|
| **Age, per 10-year increase** | 0.83 (0.55 − 1.27) | 0.381 |
| **Sex, male** | 8.89 (1.77 − 1.62×10$^{2}$) | 0.004 |
| **Body-mass index, per 1kg/m$^{2}$ increase** | 1.07 (0.95 − 1.22) | 0.267 |
| **Smoking, ever-smoker** | 9.96×10$^{6}$ (2.71 −N.E.) | 0.002 |
| **Use of statins** | 0.85 (0.23 − 2.55) | 0.789 |
| **ECOG-PS,** | | |
| **0 vs. 1** | 1.10 (0.50 − 2.46) | 0.805 |
| **0 vs. ≥2** | 3.51×10$^{6}$ (1.12 − N.E.) | 0.040 |
| **1 vs. ≥2** | 3.17×10$^{6}$ (0.99 − N.E.) | 0.051 |
| **Pathology, squamous cell (vs. non-squamous)** | 0.81 (0.35 − 1.80) | 0.604 |
| **Stage, IIIb (vs. IV/ recurrent)** | 1.15 (0.44 − 2.79) | 0.762 |
| **PD-L1 expression (TPS),** | | |
| **1 – 49% vs. <1%** | 2.34 (0.91 − 6.24) | 0.078 |
| **≥50% vs. <1%** | 7.94 (2.68 − 24.88) | <0.001 |
| **≥50% vs. 1 – 49%** | 3.39 (1.17 − 10.20) | 0.025 |
| **Treatment line, 2nd (vs. ≥3rd)** | 0.70 (0.32 − 1.52) | 0.365 |
| **LDL-cholesterol[a]** | 1.11 (0.97 − 1.25) | 0.121 |
| **HDL-cholesterol[a]** | 1.10 (0.82 − 1.48) | 0.535 |
| **Total cholesterol[a]** | 1.05 (0.96 − 1.15) | 0.258 |
| **Lauric acid[b]** | 1.15 (0.87 − 1.52) | 0.281 |
| **Myristic acid[b]** | 1.03 (0.85 − 1.23) | 0.711 |
| **Myristoleic acid[c]** | 1.00 (0.79 − 1.23) | 0.984 |
| **Palmitic acid[d]** | 1.05 (0.89 − 1.23) | 0.562 |

| | | |
|---|---|---|
| **Palmitoleic acid[b]** | 1.02 (0.93 − 1.11) | 0.628 |
| **Stearic acid[d]** | 1.25 (0.70 − 2.16) | 0.436 |
| **Oleic acid[d]** | 1.02 (0.88 − 1.17) | 0.783 |
| **Linoleic acid[d]** | 1.07 (0.91 − 1.26) | 0.415 |
| **γ-linolenic acid[c]** | 1.01 (0.95 − 1.07) | 0.689 |
| **Linolenic acid[c]** | 0.99 (0.96 − 1.02) | 0.539 |
| **Arachidic acid[c]** | 1.12 (0.96 − 1.30) | 0.159 |
| **Eicosenoic acid[c]** | 1.00 (0.84 − 1.17) | 0.998 |
| **Eicosadienoic acid[c]** | 1.10 (0.95 − 1.27) | 0.185 |
| **Eicosatrienoic acid[c]** | 1.05 (0.88 − 1.24) | 0.541 |
| **Dihomo-γ-linolenic acid[c]** | 1.02 (0.99 − 1.04) | 0.201 |
| **Arachidonic acid[b]** | 0.99 (0.91 − 1.07) | 0.845 |
| **Eicosapentaenoic aid[b]** | 0.99 (0.86 − 1.11) | 0.826 |
| **Behenic acid[c]** | 1.05 (0.98 − 1.13) | 0.157 |
| **Docosatetraenoic acid[c]** | 1.04 (0.88 − 1.21) | 0.638 |
| **Docosapentaenoic acid[c]** | 1.01 (0.95 − 1.06) | 0.764 |
| **Lignoceric acid[c]** | 1.04 (0.96 − 1.12) | 0.332 |
| **Docosahexaenoic acid[b]** | 0.95 (0.86 − 1.05) | 0.318 |
| **Nervonic acid[b]** | 1.01 (0.70 − 1.45) | 0.957 |

Data are expressed as odds ratio (95% confident interval). N.E., not estimable

ECOG-PS, Eastern Cooperative Oncology Group performance status; HDL, high-density lipoprotein; LDL, low-density lipoprotein; PD-L1, programmed cell death-ligand 1; TPS, tumor proportion score.

[a]per 10 mg/dL increase

[b]per 10 μg/mL increase

[c]per 1 μg/mL increase

[d]per 100 μg/mL increase

## Supplementary Table 9. Characteristics of the patients who received cytotoxic chemotherapy

| | n=113 |
|---|---|
| **Age, years** | 66 (61 − 72) |
| **Sex, men** | 84 (74.3) |
| **Smoking status, ever-smoker** | 88 (77.9) |
| **Body-mass index, kg/m²** | 21.9 (19.0 − 23.9) |
| **Use of statins** | 12 (10.6) |
| **ECOG-PS, 0 / 1 / ≥2** | 69 (61.1) / 36 (31.9) / 8 (7.1) |
| **Stage, IIIb / IV / recurrence** | 16 (14.2) / 97 (85.8) / 0 (0) |
| **Pathology, adeno / squamous / others** | 65 (57.5) / 31 (27.4) / 17 (15.0) |
| **PD-L1 expression: TPS, <1% / ≥1 − 49% / ≥50% / unknown** | 6 (5.3)8.5 / 3 (2.7) / 4 (3.5) / 100 (88.5) |
| ***EGFR* mutation, positive / wild / unknown** | 14 (12.4) / 98 (86.7) / 1 (0.9) |
| ***ALK* fusion gene, positive / wild / unknown** | 1 (0.9) / 73 (64.6) / 39 (34.5) |
| **Treatment line, 1st / 2nd / ≥ 3rd** | 113 (100) / 0 (0) / 0 (0) |

Data are expressed as median (interquartile range) or number (%).

ALK, anaplastic lymphoma kinase; CR, complete response; ECOG-PS, Eastern Cooperative Oncology Group performance status; EGFR, epidermal growth factor receptor; PD-1, programmed cell death-1; PD-L1, programmed cell death-ligand 1; PD, progressive disease; PR, partial response; SD, stable disease.

**Supplementary Table 10. Univariate and multivariate Cox proportional hazard analyses of progression-free survival in the chemotherapy cohort**

| Variables | Univariate | | Multivariate (single lipid analysis) | |
|---|---|---|---|---|
| | Hazard ratio | *p*-value | Hazard ratio[e] | *p*-value |
| **Age, per 10-year increase** | 1.35 (1.06 − 1.74) | 0.020 | | |
| **Sex, male** | 1.06 (0.66 − 1.78) | 0.823 | | |
| **Body-mass index, per 1kg/m$^2$ increase** | 0.98 (0.92 − 1.05) | 0.596 | | |
| **Smoking, ever-smoker** | 1.32 (0.81 − 2.28) | 0.273 | | |
| **Use of statins** | 1.11 (0.53 − 2.06) | 0.767 | | |
| **ECOG-PS,** | | | | |
| **0 vs. 1** | 0.66 (0.41 − 1.07) | 0.089 | | |
| **0 vs. ≥2** | 0.28 (0.11 − 0.96) | 0.044 | | |
| **1 vs. ≥2** | 0.43 (0.16 − 1.47) | 0.160 | | |
| **Pathology, squamous cell (vs. non-squamous)** | 1.14 (0.70 − 1.82) | 0.587 | | |
| **Stage, IIIb (vs. IV/ recurrent)** | 0.85 (0.39 − 1.61) | 0.633 | | |
| **Treatment,** | | | | |
| **cisplatin vs. carboplatin** | 0.49 (0.24 − 0.89) | 0.017 | | |
| **PEM-base vs. non-PEM / non-TAX** | 0.57 (0.30 − 1.17) | 0.119 | | |
| **TAX-base vs. non-PEM / non-TAX** | 0.73 (0.37 − 1.54) | 0.392 | | |
| **TAX-base vs. PEM-base** | 1.29 (0.79 − 2.06) | 0.300 | | |
| **LDL-cholesterol[a]** | 0.97 (0.90 − 1.04) | 0.445 | 0.99 (0.92 − 1.08) | 0.891 |
| **HDL-cholesterol[a]** | 0.95 (0.90 − 1.00) | 0.054 | 0.93 (0.76 − 1.13) | 0.496 |
| **Total cholesterol[a]** | 0.95 (0.90 − 0.99) | 0.044 | 0.97 (0.92 − 1.03) | 0.368 |
| **Lauric acid[b]** | 1.13 (0.64 − 1.73) | 0.666 | 1.17 (0.65 − 1.80) | 0.548 |
| **Myristic acid[b]** | 0.97 (0.86 − 1.06) | 0.513 | 0.98 (0.87 − 1.08) | 0.736 |
| **Myristoleic acid[c]** | 0.96 (0.84 − 1.08) | 0.555 | 0.97 (0.84 − 1.09) | 0.683 |
| **Palmitic acid[d]** | 0.98 (0.89 − 1.06) | 0.690 | 1.00 (0.91 − 1.08) | 0.991 |
| **Palmitoleic acid[b]** | 1.00 (0.95 − 1.04) | 0.870 | 0.99 (0.94 − 1.04) | 0.651 |

| | | | | |
|---|---|---|---|---|
| **Stearic acid[d]** | 0.89 (0.59 − 1.27) | 0.536 | 1.04 (0.70 − 1.49) | 0.821 |
| **Oleic acid[d]** | 0.99 (0.90 − 1.08) | 0.864 | 1.01 (0.91 − 1.10) | 0.873 |
| **Linoleic acid[d]** | 0.88 (0.79 − 0.98) | 0.018 | 0.93 (0.83 − 1.03) | 0.190 |
| **γ-linolenic acid[c]** | 0.97 (0.91 − 1.02) | 0.244 | 1.00 (0.94 − 1.04) | 0.897 |
| **Linolenic acid[c]** | 0.98 (0.96 − 1.00) | 0.080 | 0.99 (0.97 − 1.00) | 0.175 |
| **Arachidic acid[c]** | 1.00 (0.90 − 1.10) | 0.959 | 1.05 (0.94 − 1.16) | 0.381 |
| **Eicosenoic acid[c]** | 0.96 (0.85 − 1.07) | 0.530 | 0.98 (0.85 − 1.09) | 0.687 |
| **Eicosadienoic acid[c]** | 0.86 (0.74 − 0.98) | 0.037 | 0.88 (0.76 − 1.01) | 0.075 |
| **Eicosatrienoic acid[c]** | 1.01 (0.87 − 1.14) | 0.846 | 1.04 (0.90 − 1.17) | 0.546 |
| **Dihomo-γ-linolenic acid[c]** | 0.98 (0.96 − 1.00) | 0.053 | 0.99 (0.97 − 1.01) | 0.335 |
| **Arachidonic acid[b]** | 0.96 (0.91 − 1.00) | 0.046 | 0.97 (0.93 − 1.02) | 0.285 |
| **Eicosapentaenoic aid[b]** | 1.00 (0.92 − 1.06) | 0.891 | 1.01 (0.94 − 1.08) | 0.689 |
| **Behenic acid[c]** | 0.97 (0.93 − 1.01) | 0.166 | 1.01 (0.96 − 1.06) | 0.809 |
| **Docosatetraenoic acid[c]** | 0.80 (0.68 − 0.95) | 0.010 | 0.85 (0.71 − 1.01) | 0.073 |
| **Docosapentaenoic acid[c]** | 0.97 (0.94 − 1.01) | 0.132 | 0.98 (0.95 − 1.02) | 0.388 |
| **Lignoceric acid[c]** | 0.96 (0.91 − 0.99) | 0.056 | 0.99 (0.94 − 1.04) | 0.719 |
| **Docosahexaenoic acid[b]** | 0.96 (0.92 − 1.01) | 0.142 | 0.98 (0.93 − 1.02) | 0.341 |
| **Nervonic acid[b]** | 1.18 (0.93 − 1.47) | 0.151 | 1.13 (0.88 − 1.43) | 0.330 |

ECOG-PS, Eastern Cooperative Oncology Group performance status; HDL, high-density lipoprotein; LDL, low-density lipoprotein; PEM, pemetrexed; TAX, taxane.

[a]per 10 mg/dL increase

[b]per 10 μg/mL increase

[c]per 1 μg/mL increase

[d]per 100 μg/mL increase

[e]Adjusted by age, sex, ECOG-PS, and cisplatin-based therapy (vs. carboplatin-based therapy).

**Supplementary Table 11. Univariate Cox proportional hazard analyses of overall survival in the chemotherapy cohort**

| Variables | Hazard ratio | *p*-value |
|---|---|---|
| **Age, per 10-year increase** | 1.44 (1.11 − 1.88) | 0.005 |
| **Sex, male** | 1.14 (0.69 − 1.95) | 0.624 |
| **Body-mass index, per 1kg/m$^2$ increase** | 0.95 (0.89 − 1.03) | 0.226 |
| **Smoking, ever-smoker** | 2.29 (1.29 − 4.40) | 0.004 |
| **Use of statins** | 1.19 (0.53 − 2.36) | 0.647 |
| **ECOG-PS,** | | |
| **0 vs. 1** | 0.63 (0.38 − 1.06) | 0.079 |
| **0 vs. ≥2** | 0.06 (0.02 − 0.19) | <0.001 |
| **1 vs. ≥2** | 0.09 (0.03 − 0.30) | <0.001 |
| **Pathology, squamous cell (vs. non-squamous)** | 1.20 (0.70 − 1.99) | 0.502 |
| **Stage, IIIb (vs. IV/ recurrent)** | 1.19 (0.57 − 2.23) | 0.621 |
| **Treatment,** | | |
| **cisplatin vs. carboplatin** | 0.73 (0.38 − 1.29) | 0.292 |
| **PEM-base vs. non-PEM / non-TAX** | 0.63 (0.31 − 1.40) | 0.237 |
| **TAX-base vs. non-PEM / non-TAX** | 0.75 (0.36 − 1.71) | 0.479 |
| **TAX-base vs. PEM-base** | 1.20 (0.72 − 1.97) | 0.472 |
| **LDL-cholesterol[a]** | 0.93 (0.86 − 1.01) | 0.077 |
| **HDL-cholesterol[a]** | 1.05 (0.87 − 1.24) | 0.629 |
| **Total cholesterol[a]** | 0.97 (0.92 − 1.02) | 0.240 |
| **Lauric acid[b]** | 0.97 (0.89 − 1.04) | 0.391 |
| **Myristic acid[b]** | 0.89 (0.75 − 1.02) | 0.093 |
| **Myristoleic acid[c]** | 0.89 (0.74 − 1.04) | 0.167 |
| **Palmitic acid[d]** | 0.94 (0.83 − 1.05) | 0.295 |
| **Palmitoleic acid[b]** | 0.98 (0.92 − 1.04) | 0.536 |
| **Stearic acid[d]** | 0.75 (0.45 − 1.17) | 0.214 |

| | | |
|---|---|---|
| **Oleic acid[d]** | 0.96 (0.85 − 1.07) | 0.498 |
| **Linoleic acid[d]** | 0.95 (0.85 − 1.06) | 0.396 |
| **γ-linolenic acid[c]** | 1.00 (0.94 − 1.04) | 0.914 |
| **Linolenic acid[c]** | 0.99 (0.97 − 1.01) | 0.388 |
| **Arachidic acid[c]** | 0.98 (0.87 − 1.09) | 0.739 |
| **Eicosenoic acid[c]** | 0.98 (0.85 − 1.11) | 0.780 |
| **Eicosadienoic acid[c]** | 0.94 (0.81 − 1.08) | 0.409 |
| **Eicosatrienoic acid[c]** | 1.05 (0.89 − 1.20) | 0.509 |
| **Dihomo-γ-linolenic acid[c]** | 1.00 (0.98 − 1.02) | 0.986 |
| **Arachidonic acid[b]** | 1.02 (0.97 − 1.07) | 0.397 |
| **Eicosapentaenoic aid[b]** | 1.03 (0.95 − 1.10) | 0.437 |
| **Behenic acid[c]** | 0.97 (0.92 − 1.02) | 0.207 |
| **Docosatetraenoic acid[c]** | 1.02 (0.86 − 1.20) | 0.852 |
| **Docosapentaenoic acid[c]** | 0.99 (0.96 − 1.03) | 0.768 |
| **Lignoceric acid[c]** | 0.96 (0.92 − 1.01) | 0.113 |
| **Docosahexaenoic acid[b]** | 1.00 (0.95 − 1.05) | 0.873 |
| **Nervonic acid[b]** | 1.26 (0.99 − 1.60) | 0.064 |

ECOG-PS, Eastern Cooperative Oncology Group performance status; HDL, high-density lipoprotein; LDL, low-density lipoprotein; PEM, pemetrexed; TAX, taxane.

[a]per 10 mg/dL increase

[b]per 10 μg/mL increase

[c]per 1 μg/mL increase

[d]per 100 μg/mL increase

**Supplementary Table 12. Univariate logistic regression analyses of objective response in the chemotherapy cohort**

| Variables | Odds ratio | *p*-value |
|---|---|---|
| **Age, per 10-year increase** | 0.68 (0.45 − 1.02) | 0.056 |
| **Sex, male** | 1.08 (0.46 − 2.53) | 0.854 |
| **Body-mass index, per 1kg/m$^2$ increase** | 1.02 (0.91 − 1.14) | 0.743 |
| **Smoking, ever-smoker** | 1.27 (0.52 − 3.10) | 0.596 |
| **Use of statins** | 0.74 (0.22 − 2.46) | 0.625 |
| **ECOG-PS,** | | |
| **0 vs. 1** | 1.24 (0.55 − 2.81) | 0.599 |
| **0 vs. ≥2** | 4.67 (0.88 − 24.83) | 0.071 |
| **1 vs. ≥2** | 3.75 (0.66 − 21.15) | 0.134 |
| **Pathology, squamous cell (vs. non-squamous)** | 0.76 (0.33 − 1.73) | 0.508 |
| **Stage, IIIb (vs. IV/ recurrent)** | 0.55 (0.19 − 1.59) | 0.266 |
| **Treatment,** | | |
| **cisplatin vs. carboplatin** | 0.92 (0.35 − 2.44) | 0.871 |
| **PEM-base vs. non-PEM / non-TAX** | 0.96 (0.30 − 3.04) | 0.945 |
| **TAX-base vs. non-PEM / non-TAX** | 0.70 (0.21 − 2.38) | 0.572 |
| **TAX-base vs. PEM-base** | 0.73 (0.32 − 1.67) | 0.459 |
| **LDL-cholesterol[a]** | 1.08 (0.96 − 1.22) | 0.203 |
| **HDL-cholesterol[a]** | 0.91 (0.69 − 1.20) | 0.523 |
| **Total cholesterol[a]** | 1.02 (0.94 − 1.10) | 0.646 |
| **Lauric acid[b]** | 1.01 (0.92 − 1.10) | 0.839 |
| **Myristic acid[b]** | 1.04 (0.89 − 1.23) | 0.611 |
| **Myristoleic acid[c]** | 1.06 (0.87 − 1.29) | 0.584 |
| **Palmitic acid[d]** | 1.01 (0.89 − 1.15) | 0.864 |
| **Palmitoleic acid[b]** | 1.01 (0.94 − 1.09) | 0.738 |
| **Stearic acid[d]** | 0.80 (0.44 − 1.44) | 0.447 |

| | | |
|---|---|---|
| **Oleic acid[d]** | 0.98 (0.85 − 1.12) | 0.740 |
| **Linoleic acid[d]** | 0.96 (0.82 − 1.13) | 0.652 |
| **γ-linolenic acid[c]** | 0.99 (0.94 − 1.04) | 0.647 |
| **Linolenic acid[c]** | 1.00 (0.97 − 1.04) | 0.923 |
| **Arachidic acid[c]** | 1.03 (0.87 − 1.22) | 0.764 |
| **Eicosenoic acid[c]** | 0.95 (0.78 − 1.15) | 0.585 |
| **Eicosadienoic acid[c]** | 0.93 (0.75 − 1.16) | 0.517 |
| **Eicosatrienoic acid[c]** | 0.89 (0.73 − 1.09) | 0.243 |
| **Dihomo-γ-linolenic acid[c]** | 0.98 (0.96 − 1.01) | 0.280 |
| **Arachidonic acid[b]** | 0.95 (0.88 − 1.01) | 0.108 |
| **Eicosapentaenoic aid[b]** | 0.95 (0.84 − 1.08) | 0.451 |
| **Behenic acid[c]** | 1.03 (0.96 − 1.11) | 0.405 |
| **Docosatetraenoic acid[c]** | 0.80 (0.63 − 1.04) | 0.087 |
| **Docosapentaenoic acid[c]** | 0.98 (0.92 − 1.03) | 0.416 |
| **Lignoceric acid[c]** | 1.02 (0.95 − 1.10) | 0.580 |
| **Docosahexaenoic acid[b]** | 0.97 (0.89 − 1.05) | 0.429 |
| **Nervonic acid[b]** | 0.77 (0.49 − 1.19) | 0.230 |

[a]per 10 mg/dL increase

[b]per 10 μg/mL increase

[c]per 1 μg/mL increase

[d]per 100 μg/mL increase
